# Characterization of methicillin-resistant *Staphylococcus aureus* through genomics approach

**DOI:** 10.1007/s13205-020-02387-y

**Published:** 2020-08-20

**Authors:** Romen Singh Naorem, Peter Urban, Gunajit Goswami, Csaba Fekete

**Affiliations:** 1 Department of General and Environmental Microbiology, Institute of Biology, University of Pécs, Pécs, 7624 Hungary; 2 Microbial Biotechnology Research Group, Szentágothai Research Centre, Pécs, 7624 Hungary; 3 Department of Life Sciences, Dibrugarh University, Dibrugarh, 786004 Assam India

**Keywords:** MRSA, Molecular typing, Whole genome sequencing, Comparative genome analysis

## Abstract

In the present study, a total of 35 *S. aureus* isolates collected from two different geographical locations viz., Germany and Hungary were tested for their methicillin-resistant phenotype which revealed a high incidence of methicillin-resistant *S. aureus*. The quantitative test for biofilm production revealed that 73.3% of isolates were biofilm producers. The isolates were further characterized using a set of biochemical and genotypic methods such as amplification and analysis of *S. aureus* species-specific sequence and *mecA* gene. The 33 *mecA* positive isolates were then characterized by the amplification of *SCCmec* and *pvl* toxin genes. Further, based on the biofilm-forming phenotype, 15 isolates were selected and characterized through PCR–RFLP of *coa* gene, polymorphism of *spa* gene and amplification of biofilm-associated genes. The dendrogram prepared from the results of both biochemical and genotypic analyses of the 15 isolates showed that except for the isolates SA G5 and SA H29, the rest of the isolates grouped themselves according to their locations. Thus, the two isolates were selected for further characterization through whole-genome sequencing. Comparative genome analysis revealed that SA G5 and SA H29 have 97.20% ANI values with 2344 gene clusters (core-genome) of which 16 genes were related to antibiotic resistance genes and 57 genes encode virulence factors. The highest numbers of singleton genes were found in SA H29 that encodes proteins for virulence, resistance, mobile elements, and lanthionine biosynthesis. The high-resolution phylogenetic trees generated based on shared proteins and SNPs revealed a clear difference between the two strains and can be useful in distinguishing closely related genomes. The present study demonstrated that the whole-genome sequence analysis technique is required to get a better insight into the MRSA strains which would be helpful in improving diagnostic investigations in real-time to improve patient care.

**Electronic supplementary material:**

The online version of this article (10.1007/s13205-020-02387-y) contains supplementary material, which is available to authorized users.

## Introduction

*Staphylococcus aureus* is one of the leading causes in both communities- and nosocomial-acquired infections. It acquires an arsenal of antibiotic resistance genes (ARGs) and virulence factors-encoding genes (VFGs) that are subjected to horizontal gene transfer (HGT) and recombination (Hughes and Friedman 2005; Chan et al. 2011). It can cause a diverse range of infections including chronic skin and soft tissue infections to life-threatening illnesses (Stefani and Varaldo 2003; Yamamoto et al. 2013; Mottola et al. 2016). The genomic plasticity of *S. aureus* has enabled the emergence of hypervirulent and drug-resistant strains and led to challenging issues in antibiotic therapy. Consequently, the morbidity and mortality rates caused by *S. aureus* infections have a substantial impact on health concern (Denis 2017). Methicillin-resistant *S. aureus* (MRSA) acquired methicillin resistance gene (*mecA*) which is present within Staphylococcal Chromosomal Cassette *mec* (*SCCmec*) (Zhang et al. 2012) and reduced affinity of penicillin and β-lactam antibiotics (Jansen et al. 2006; Mistry et al. 2016). *S. aureus* anchors epithelial surfaces and produces biofilm (Strandén et al. 2003; Goudarzi et al. 2017). Biofilms act as a barrier against many antibiotics and other stressors and prevent their entry into the cells. Biofilms also defend the bacterial cells from host-immune evasion (Mah and O’Toole 2001; Donlan and Costerton 2002; Le et al. 2018; Vestby et al. 2020). Biofilm is a complex 3D structure of sessile microbial community covered by an exopolysaccharide glycocalyx (Otto 2008; Deka et al. 2019). The biofilm-forming ability depends on several physicals, chemical, and biological factors (Garrett et al. 2008). The biofilm-forming processes of *S. aureus* are determined by the *icaADBC* gene cluster, responsible for the synthesis of polysaccharide intracellular adhesin (PIA), and capsular polysaccharide/adhesion (PS/A) (Chaieb et al. 2005; Arciola et al. 2015; Hoang et al. 2019). The PIA is composed of β-1,6-linked N-acetylglucosamine with partially deacetylated residues, a major component of the exopolysaccharide matrix that surrounds bacterial cells in the biofilm (Mack et al. 1996; Vuong et al. 2004). It was reported that the co-existence of *icaA* and *icaD* increase N-acetylglucosaminyltransferase activity and slime production (Arciola et al. 2001, 2006). Additionally, *S. aureus* possesses microbial surface components recognizing adhesive matrix molecules (MSCRAMMs), such as elastin (*ebps*), laminin (*eno*), collagen (*cna*), fibronectin A and B (*fnbA* and *fnbB*), fibrinogen (*fib*), bone sialoprotein (*bbp*) and clumping factors A and B (*clfA* and *clfB*) and these molecules are present on the bacterial surface to enable adherence to host tissues, thus playing a pivotal role in pathogenesis (Lindsay et al. 2006; Foster et al. 2014; Ghasemian et al. 2015; Dufrêne and Viljoen 2020).

Methods for molecular typing of MRSA that depend on gene-specific polymerase chain reaction such as *SCCmec*, *pvl* (Panton-Valentine leukocidin), *coa* (coagulase), and *spa* (*S. aureus* protein A) genes and followed by restriction enzyme digestion (PCR–RFLP) has proven its good discriminatory power (DP) and is used routinely for typing MRSA strains (Faria et al. 2008; Omar et al. 2014; Al-Obaidi et al. 2018; Tenover et al. 2019; Alkharsah et al. 2019). *SCCmec* typing classifies the MRSA into hospital-associated (HA-MRSA) and community-associated (CA-MRSA) strains (Appelbaum 2007). HA-MRSA isolates carried *SCCmec* types I to III, but CA-MRSA isolates has a novel, small variant of *SCCmec* IV and V, but also has the locus for *pvl* gene (Baba et al. 2002; Shukla et al. 2012). The coagulase enzyme, a virulence factor encoded by the *coa* gene contains several tandem repeats suitable to generate polymorphic RFLP patterns among different isolates. These molecular typing methods may be helpful in determining the relatedness among geographically diverse MRSA (Singh et al. 2006; Grundmann et al. 2010).

Phenotypic analysis including the antibiotic resistance patterns and molecular typing methods are beneficial for identifying the risk factors associated with MRSA infections which support the establishment of adequate infection control programs (Zhang et al. 2012; Mistry et al. 2016). Epidemiological studies of MRSA apply various molecular typing techniques such as Pulsed-Field Gel Electrophoresis (PFGE), *SCCmec*, *spa* genes typing, Multi-Locus Sequence Typing (MLST), and detection of *pvl* gene as well as PCR–RFLP of *coa* gene (Zhang et al. 2012; Al-Obaidi et al. 2018). Many of these established techniques are costly and time-consuming, and the discriminatory abilities of these techniques are also different (Du et al. 2011). However, the *spa* typing method has been considered as a rapid and inexpensive method for genotyping and it provides high discriminating power than other typing methods (Shittu et al. 2011; O’Hara et al. 2016; Goudarzi et al. 2017; Ali et al. 2019; Rezai et al. 2019; Kareem et al. 2020). Both phenotypic and molecular typing methods have been used widely to detect and differentiate several MRSA strains, but these techniques have certain limitations in infection control and investigating the nosocomial transmission as these techniques provide low resolution and more time-consuming. Because of that in recent times whole genome-based typing has been used as it offers an excellent resolution in global and local epidemiological investigations of pathogen outbreaks and offers further data mining activities essentially for ARGs and VFGs profiling (Köser et al. 2012). So, the Next Generation Sequencer (NGS) based-genome sequencing technique has become a vital tool in the clinical microbiology arenas for comparative genomic analysis of several other species of the Staphylococcus genus in terms of the niche adaptation, combat antibiotics, and emergence of new virulent strains in real-time (Al-Obaidi et al. 2018; McClure et al. 2018; Tenover et al. 2019; Maljkovic Berry et al. 2019; Raven et al. 2020). In the current study, we performed characterization through molecular typing methods and their integrated polyphasic approach to determine the *S. aureus* lineage strains. The lineage strains were compared deeply into a genomic level based on Average Nucleotide Identity (ANI), genome distance, orthologue gene/ clusters, and the evolutionary relationship. Further, ARGs and VFGs were characterized to understand their crucial role in pathogenesis and defense. The information on genomic characteristics and comparative genomics of *S. aureus* will facilitate investigations into the molecular basis of pathogenesis and improve diagnostic investigations of infectious diseases in real-time and improve patient care.

## Materials and methods

### Collection and preliminary identification of the isolates

In this study, 35 *S. aureus* strains were collected from the Department of Medical Microbiology and Immunology Laboratory, Medical School, University of Pecs, Hungary. The Hungarian *S. aureus* strains (60%) were previously recovered during February to July 2016 from wounds (31.42%), blood (8.57%), tracheas (5.71%), *ear*s (2.85%), lungs (2.85%), nostrils (2.85%), skins (2.85%) and throats (2.85%) while the German *S. aureus* strains (40%) were recovered from body sites without documentation. The isolates were identified as Staphylococcal strain based on colony morphology on Nutrient agar, Blood Agar, Mannitol Salt Agar, Gram staining, and different biochemical tests (Bergey and Holt 1994). The isolates were tested for catalase, coagulase, urease, DNase production, and mannitol fermentation test (Collee et al. 1996).

### Antibiotic susceptibility test

The 35 *S. aureus* clinical strains were screened for MRSA using BBL™ CHROMagar™ MRSA II media (BD, USA). Susceptibility of *S. aureus* strains to oxacillin (1 μg), cefoxitin (30 μg), erythromycin (15 μg) and vancomycin (30 μg) were determined using disk diffusion method according to Clinical and Laboratory Standards Institute (CLSI) guidance (CLSI 2014). The entire antimicrobial susceptibility test (AST) was repeated three times using the *S. aureus* ATCC25923 and ATCC700698 as MRSA negative and positive controls, respectively. The diameter zone of inhibition was measured in millimetre (mm).

### Biofilm formation assay

Biofilm formation was performed as previously described (Rahimi et al. 2016) with some modifications. Briefly, *S. aureus* strains were cultured overnight at 37 °C in tryptic soy broth (TSB) (BD, Germany) containing 0.25% (w/v) glucose. The cell density was adjusted to a final concentration of 10^6^ CFU/ml in TSB supplemented with 0.25% (w/v) glucose. Cell suspensions (200 µl) were loaded into 96-well round-bottomed microtiter plate (Sarstedt, Germany), and incubated at 37 °C for 18 h without shaking. Cells were washed three times with 200 µl sterile PBS (pH 7.2), dried at room temperature and fixed with methanol (99% v/v). The dried biofilm was stained with 200 µl of 0.16% (w/v) crystal violet for 15 min. To remove the unbound dye, biofilms were washed three times with PBS and air dry. Finally, the biofilm-bound dye was solubilized with 200 µl of 95% (v/v) ethanol, and absorbance was measured at 540 nm wavelength using a Multiskan Ex microtiter plate reader (Thermo Electron Corporation, USA) in a flat-bottom 96-well plate (Costar 3599; Corning; USA). Experiments were performed in triplicates with *S. aureus* ATCC25923 as a biofilm-positive control strain.

### Molecular identification and genotyping

The DNA of the 35 strains was extracted from the overnight culture of *S. aureus* using QIAamp DNA Mini Kit (Qiagen GmbH, Hilden, Germany). The extracted DNA concentration was assayed by the Nanodrop-2000 spectrophotometer. Genomic DNA was used for the detection of *S. aureus* species-specific sequence (Martineau et al. 1996), *mecA* (Strommenger et al. 2003), and the *pvl* toxin (Hisata et al. 2005; Karahan and Çetinkaya 2007) genes. *S. aureus* ATCC25923, ATCC700698, and ATCC700699 strains were used as reference strains for *mecA* negative and positive controls respectively. Multiplex PCR typing of *SCCmec* gene was performed on *mecA*-positive *S. aureus* strains using primers as described previously (Zhang et al. 2005). The MRSA isolates that showed unexpected amplified fragments or no amplification were defined as non-typeable (NT). For PCR–RFLP, the *coa* gene amplicons were digested with HaeIII (Fermentas, USA) restriction enzyme (Khoshkharam-Roodmajani et al. 2014) and a heat-map with dendrogram was generated from the restriction banding pattern using Morpheus web-based program (https://software.broadinstitute.org/morpheus/) using the Euclidean distance feature. Polymorphism of the *spa* gene was detected based on a previously described primer set (Harmsen et al. 2003). The PCR products were purified using the ZR-96 DNA Clean-up Kit (Zymo Research, USA). Concentration was determined by Qubit 3.0 and sequencing reactions were performed using BigDye Terminator v3.1 Cycle Sequencing Kit (Applied Biosystems, USA). Sequencing reactions were run on ABI PRISM 310 Genetic Analyzer (Applied Biosystems, USA). The *spa* sequence types were assigned using spaTyper (https://spatyper.fortinbras.us/) and confirmed by a *spa* database (https://spa.ridom.de/) in DNAGear software (Al-Tam et al. 2012). The *spa* sequences were aligned, and a phylogenetic tree was constructed by the UPGMA method in MEGA X software (Kumar et al. 2018). The DPs of *coa* and *spa* typing were calculated based on Simpson's index using the DP calculator, available online (https://insilico.ehu.es/mini_tools/discriminatory_power/).

Biofilm-encoding genes were amplified using the primer sets listed in Supplementary Table 2. The PCR amplification was performed using DreamTaq PCR Master Mix according to the manufacturer's recommendation (Thermo Fisher Scientific, USA) in a Veriti™ 96-Well Thermal Cycler (Applied Biosystem, USA) as follows: 96 °C for 3 min; 96 °C for 30 s, 54 °C for 30 s, 72 °C for 1 min repeated for 35 cycles; final extension was performed at 72 °C for 7 min. The amplified products were electrophorized on 2% (w/v) agarose gel, stained using 0.5 µg/ml ethidium bromide solution, and captured using the FluroChem Q system (ProteinSimple™, USA).

### Data setting for a polyphasic approach

Individual result of the applied techniques was converted into the unweighted binary code (0, 1), Jacquard’s similarity index was generated and visualized according to the Neighbour-Joining (NJ) clustering method using Past 3.x (Hammer et al. 2001). Besides the binary data (Supplementary file 2) was used to perform a logistic Principal Component Analysis (PCA) in R software.

### Whole-genome sequencing

**Fig. 1:**
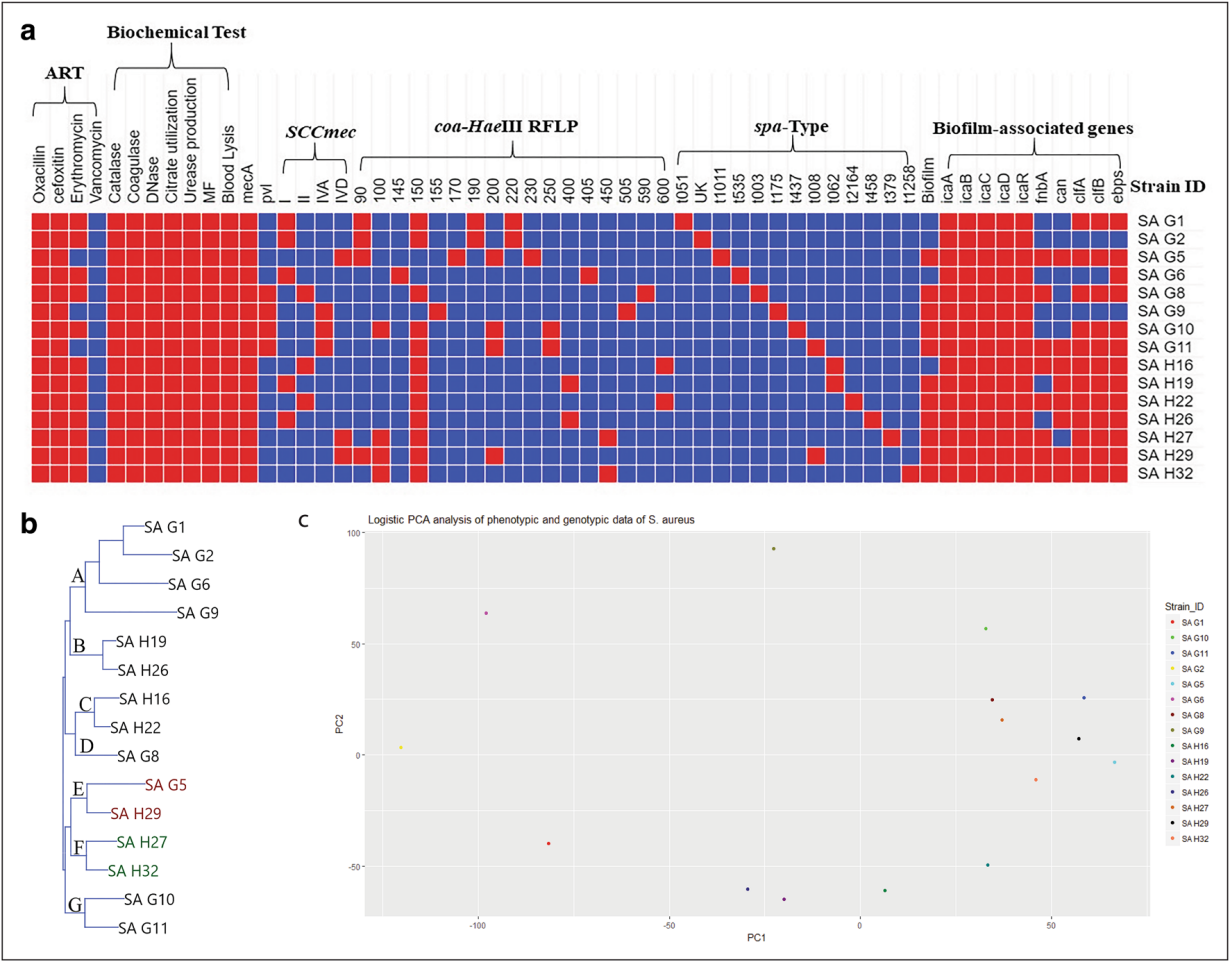
Polyphasic comparison of *S. aureus* clinical isolates. **a** Colored similarity matrix categories representing resistant test against different antibiotics (ART), biochemical test, PCR based *mecA* and *pvl* genes detection, *SCCmec* typing, *coa*-HaeIII RFLP, *spa* type, biofilm-forming assay and detection of biofilm-associated genes. Red and blue colors indicate the presence ( +) and absence (−) of particular properties respectively. **b** NJ-dendrogram prepared from the results of biochemical and molecular analysis of the 15 *S. aureus* clinical isolates (SA). The isolates were labeled according to their geographical origin where G and H indicate Germany and Hungary. Clusters are labeled as A-F. **c** Principal component analysis of the results of biochemical and molecular analysis of the 15 *S. aureus* clinical isolates

Based on the dendrogram and PCA plot generated from combined results of the phenotypic and genotypic analysis (binary data) of the *S. aureus* strains it was observed that two strains viz., SA G5 (collected from Germany) and SA H29 (collected from Hungary) were found in the same group (Fig. 1a–c). Therefore, these two strains (SA G5 and SA H29) were selected for further analysis through whole-genome sequencing to get a better insight into their genomic background.

### Genomic DNA extraction, library preparation, and sequencing

Whole-genome sequencing was performed at Microbial Biotechnology Research Group, Szentágothai Research Centre, University of Pecs, Hungary with the following procedures: the genomic DNA was extracted using the GenElute™ Bacterial Genomic DNA Kit (Sigma, USA) following the manufacturer’s recommendation. The extracted DNA samples were quantified in a Qubit 3.0 fluorometer (Invitrogen, USA) using dsDNA High Sensitivity (HS) Assay Kit (Thermo Fisher Scientific Inc. USA) and subsequently, DNA quality was visualized by agarose gel electrophoresis. Genomic libraries were prepared using the NEB Next Fast DNA Fragmentation and Library Preparation Kit, developed for Ion Torrent (New England Biolabs, USA) and used according to 200 bp protocol. After chemical fragmentation, DNA size selection was performed on precast 2% E-Gel Size Select Gel (Thermo Fisher Scientific Inc. USA). The quality of the libraries was verified using Agilent high sensitivity DNA assay kit (Agilent Technologies Inc. USA) in Agilent 2100 Bioanalyzer System (Agilent Technologies Inc. USA). For the template preparation, Ion PGM Hi-Q View OT2 Kit was used (Thermo Fisher Scientific Inc. USA). The template positive beads were loaded on Ion 316v2 Chip and sequenced using Ion PGM Hi-Q View Sequencing Kit on Ion Torrent Personal Genome Machine (PGM) (Thermo Fisher Scientific Inc. USA).

### Genome assembly, annotation, and comparison

Quality trimming of the reads was performed with the Ion Torrent Suite 5.4.0 (Thermo Fisher Scientific Inc. USA) with default settings. De novo genome assembly was performed by SPAdes 3.7.1 software with default parameters for Ion Torrent data and *k-mer* settings of 21, 33, 55, 77, 99, and 127 (Nurk et al. 2013). Closely related reference genomes were identified using kmerFinder 3.1 and NCBI microbial genome blast web-platforms. Identified high-quality reference genomes, CP006044.1 (*S. aureus* CA-347), and CP023390 (*S. aureus* subsp*. aureus str. Newman*) were applied to orientate and order contigs of SA G5 and SA H29 respectively using the ‘Move Contigs’ algorithm in Mauve 2.4.0 (Darling et al. 2010). Scaffolds were generated using the reference-based scaffolder MeDuSa (Bosi et al. 2015). Gene annotation was performed by two independent automated pipelines based on Rapid Annotation using Subsystem Technology (RAST) (Aziz et al. 2008) and NCBI Prokaryotic Genome Annotation Pipeline (Tatusova et al. 2016).

The values for ANI were calculated using the OrthoANIu algorithm (Yoon et al. 2017). Also, dDDH (digital DNA-DNA Hybridization) estimates were obtained from Genome to Genome Distance Calculator 2.1 (Meier-Kolthoff et al. 2013). RAST server-based SEED viewer was used for the subsystem functional categorization (Overbeek et al. 2014). The protein-coding sequence identified by RAST was analyzed for orthologous genes/clusters and subsequent functional annotation using web platform OrthoVenn2 (Xu et al. 2019). Graphical map of sequence features embedded circular genomes of SA G5, CP006044.1, SA H29, and CP023390.1 were generated using CGView Server (Grant and Stothard 2008) with default parameters against the reference genome *Staphylococcus aureus subsp. aureus* NCTC 8325 (NC_007795.1). ARGs and VFGs were identified by the Comprehensive Antibiotic Resistance Database (CARD) and the virulence factor database (VFDB) in PATRIC 3.5.43 (Chen et al. 2016; Wattam et al. 2017). The genome assemblies were screened for plasmid replicon (*rep*) genes in nonaligned contigs or scaffold regions using PlasmidFinder version 2.1 (Carattoli et al. 2014) with default parameters. The identified nonaligned contigs or scaffolds associated with plasmid sequences were extracted and used for the identification of full-length plasmid regions using PLSDB (Plasmid Database) version-2020-03-04 with search strategy Mash screen, and the default values were a maximum *P* value of 0.1 and a minimum identity of 0.99 (Galata et al. 2019).

### Phylogenetic analysis

The 16S rRNA sequences obtained from the whole genome data of the two strains were aligned with the other closely related 16S rRNA sequences of *S. strains* and *Bacillus cereus* ATCC14579 (AE016877.1) using MUSCLE algorithm in MEGA X (Kumar et al. 2018). The phylogenetic tree was then constructed by the NJ method with 500 replicates. Phylogenetic trees were generated based on the concatenated alignment of all shared proteins (core) of *S. aureus* genome sequences with *B. cereus* ATCC14579 (AE016877.1) as outgroup by RAxML in PATRIC 3.5.43 (Wattam et al. 2017). SNPs were filtered from the genome sequences and generate phylogeny based on the concatenated alignment of the high-quality SNPs using CSI phylogeny 1.4 servers (https://cge.cbs.dtu.dk/services/CSIPhylogeny/) and the tree was visualized in MEGA X (Kumar et al. 2018).

### Sequence supporting data

The genomic data of this study were deposited in the NCBI genome database under the GenBank accession numbers: CP032160 and CP032468-CP032470.

## Results

### Phenotypic characterization

The colony characteristics of the strain are yellow colored, moist, round, glistening opaque colonies with β or weak hemolysis on blood agar. The strains are Gram-positive cocci showing typical staphylococcal bunch.

### Antibiotic susceptibility test

The 35 *S. aureus* clinical strains were screened for MRSA using BBL™ CHROMagar™ MRSA II media which revealed that 94.28% of the strains were MRSA. The tested strains were highly resistant to beta-lactam antibiotics viz*.* oxacillin (94.28%) and cefoxitin (94.28%), but less resistant to non-beta lactam antibiotics, erythromycin (71.43%), and none of the strain displayed resistant to vancomycin (Table 1).

**Table 1:** Antibiotic susceptibility patterns of *S. aureus* isolates

Strain ID	Origin	Diameter of inhibition zone (mm)			
		Ox-1 µg	Cfox-30 μg	Ery-15 µg	Van-30 μg
SA G1	HA	6 ± 0 (R)	6 ± 0 (R)	6 ± 0 (R)	19.5 ± 0.7 (S)
SA G2	HA	11.5 ± 0.7 (R)	6 ± 0 (R)	6 ± 0 (R)	20 ± 0.7 (S)
SA G3	HA	28 ± 0 (S)	18.5 ± 0.7 (S)	27 ± 0 (S)	21 ± 1.4 (S)
SA G4	HA	6 ± 0 (R)	6 ± 0 (R)	24.5 ± 7.7 (S)	20.5 ± 0.7 (S)
SA G5	HA	6 ± 0 (R)	6 ± 0 (R)	24.5 ± 7.7 (S)	19.5 ± 0.7 (S)
SA G6	HA	6 ± 0 (R)	6 ± 0 (R)	6 ± 0 (R)	20 ± 0 (S)
SA G7	HA	24.5 ± 0.7 (S)	18 ± 0 (S)	30 ± 0 (S)	18.5 ± 0.7 (S)
SA G8	HA	6 ± 0 (R)	6 ± 0 (R)	6 ± 0 (R)	18.5 ± 0.7 (S)
SA G9	CA	6 ± 0 (R)	6 ± 0 (R)	24 ± 8 (S)	18 ± 0 (S)
SA G10	CA	6 ± 0 (R)	6 ± 0 (R)	6 ± 0 (R)	20 ± 1.4 (S)
SA G11	CA	6 ± 0 (R)	6 ± 0 (R)	10 ± 0 (S)	19 ± 1.4 (S)
SA G12	CA	6 ± 0 (R)	7.5 ± 2.1 (R)	30 ± 0 (S)	19.5 ± 07 (S)
SA G13	CA	6 ± 0 (R)	6 ± 0 (R)	30 ± 0 (S)	19 ± 0 (S)
SA G14	CA	6 ± 0 (R)	6 ± 0 (R)	30 ± 0 (S)	19 ± 0 (S)
SA H15	HA	6 ± 0 (R)	6 ± 0 (R)	6 ± 0 (R)	19 ± 0 (S)
SA H16	HA	6 ± 0 (R)	6 ± 0 (R)	6 ± 0 (R)	20 ± 0 (S)
SA H17	HA	6 ± 0 (R)	6 ± 0 (R)	6 ± 0 (R)	18 ± 0 (S)
SA H18	HA	6 ± 0 (R)	6 ± 0 (R)	6 ± 0 (R)	18.5 ± 0.7 (S)
SA H19	HA	6 ± 0 (R)	6 ± 0 (R)	6 ± 0 (R)	19.5 ± 0.7 (S)
SA H20	HA	6 ± 0 (R)	6 ± 0 (R)	6 ± 0 (R)	19 ± 1.4 (S)
SA H21	HA	6 ± 0 (R)	6 ± 0 (R)	11 ± 0 (R)	18.5 ± 0.7 (S)
SA H22	HA	6 ± 0 (R)	6 ± 0 (R)	11 ± 0 (R)	18.5 ± 0.7 (S)
SA H23	HA	6 ± 0 (R)	7.5 ± 2.1 (R)	6 ± 0 (R)	20 ± 0 (S)
SA H24	HA	6 ± 0 (R)	6 ± 0 (R)	6 ± 0 (R)	19 ± 0 (S)
SA H25	HA	6 ± 0 (R)	6 ± 0 (R)	6 ± 0 (R)	20 ± 0 (S)
SA H26	HA	8 ± 2.8 (R)	14.5 ± 0.7 (R)	6 ± 0 (R)	19 ± 1.4 (S)
SA H27	HA	6 ± 0 (R)	6 ± 0 (R)	16.14 ± 0 (R)	19.5 ± 0.7 (S)
SA H28	HA	6 ± 0 (R)	6 ± 0 (R)	24.5 ± 0.7 (R)	19 ± 1.4 (S)
SA H29	HA	10.5 ± 0.7 (R)	8 ± 2.8 (R)	6 ± 0 (R)	19 ± 1.4 (S)
SA H30	HA	6 ± 0 (R)	3 ± 0 (R)	6 ± 0 (R)	18.5 ± 2.1 (S)
SA H31	HA	6 ± 0 (R)	6 ± 0 (R)	6 ± 0 (R)	18.5 ± 0.7 (S)
SA H32	HA	6 ± 0 (R)	6 ± 0 (R)	6 ± 0 (R)	19 ± 1.4 (S)
SA H33	HA	6 ± 0 (R)	6 ± 0 (R)	6 ± 0 (R)	19 ± 1.4 (S)
SA H34	HA	6 ± 0 (R)	6 ± 0 (R)	6 ± 0 (R)	19.5 ± 0.7 (S)
SA H35	HA	6 ± 0 (R)	6 ± 0 (R)	6 ± 0 (R)	19 ± 1.4 (S)
ATCC700698	–	6 ± 0 (R)	6 ± 0 (R)	6 ± 0 (R)	19.5 ± 0.7 (S)
ATCC25923	–	26.5 ± 0.7 (S)	25 ± 0 (S)	33.5 ± 0.7 (S)	20.5 ± 0.7 (S)

Zone of inhibition in mm is given as Mean ± SD; *R *and* S* denote the resistant and susceptible, *Ox* oxacillin (≤ 10 mm = Resistant), *Cfox* cefoxitin (≤ 14 mm = Resistant), *Ery* erythromycin (≤ 13 mm = Resistant), *Van* vancomycin (≤ 9 mm = Resistant)

^a^SA G and SA H represent strains isolated from Germany and Hungary respectively. HA and CA represent the Hospital and Community-associated *S. aureus* strains respectively

### Biofilm production test

The quantitative test for biofilm production revealed that among the 33 methicillin-resistant strains, 87.87% of strains produced biofilm. Fifteen strains were selected among 33 methicillin-resistant strains based on non- (26.7%), moderate- (40%), and strong- (33.3%) biofilm-forming abilities for molecular typing and discrimination. (Supplementary Table 1).

### Molecular characterization and genotyping

The PCR amplified products showed that 100% strains of *S. aureus* were positive to *S. aureus* species-specific sequence represented by a distinct band of 107 base pairs (bp) (Supplementary Fig. 1). The *mecA* and *pvl* genes were detected in 94.28% with an amplicon size of 532 bp and 22.85% with the amplicon size 1918 bp respectively (Supplementary Figs. 2, 3). The AST and *mecA* gene detection revealed concordant results, thus 94.28% of *S. aureus* isolates were confirmed as MRSA.

*SCCmec* typing revealed that *SCCmec* type II (33.33%) was the most predominant followed by type I (21.21%), type IVa (15.5%), type IVd (9.09%), IVb (3.03%) and the rest of the strains were non-typeable (12.12%). Among the 35 strains, eight strains were positive for *pvl* of which four strains belonged to *SCCmec* II, three strains to *SCCmec* IV and, one to *SCCmec* V (Supplementary Fig.4). The distribution of *SCCmec* types showed that 36.36% of strains were CA-MRSA and 54.54% strains were HA-MRSA (Table 2).

**Table 2:** Biochemical tests and PCR based molecular detection of *mecA*, *pvl* and *SCCmec* genes of *S. aureus* isolates

^a^Strain ID	Biochemical tests								Molecular detection		
	Catalase	Coagulase	DNase	Citrate utilization	Urease production	MF	*Blood Lysis*	Biofilm	*mecA*	*pvl*	*SCCmec* type
SA G1	+	+	+	+	+	+	+	−	+	−	I
SA G2	+	+	+	+	+	+	+	−	+	−	I
SA G3	+	+	+	+	+	+	+	+	−	−	NT
SA G4	+	+	+	+	+	+	+	+	+	−	NT
SA G5	+	+	+	+	+	+	+	+	+	−	IVd
SA G6	+	+	+	+	+	+	+	−	+	−	I
SA G7	+	+	+	+	+	+	+	+	−	−	NT
SA G8	+	+	+	+	+	+	+	+	+	+	II
SA G9	+	+	+	+	+	+	+	+	+	+	IVa
SA G10	+	+	+	+	+	+	+	+	+	+	IVa
SA G11	+	+	+	+	+	+	+	+	+	+	IVa
SA G12	+	+	+	+	+	+	+	+	+	−	IVa
SA G13	+	+	+	+	+	+	+	+	+	−	IVa
SA G14	+	+	+	+	+	+	+	+	+	+	V
SA H15	+	+	+	+	+	+	+	+	+	−	II
SA H16	+	+	+	+	+	+	+	−	+	+	II
SA H17	+	+	+	+	+	+	+	+	+	−	II
SA H18	+	+	+	+	+	+	+	+	+	−	I
SA H19	+	+	+	+	+	+	+	+	+	−	I
SA H20	+	+	+	+	+	+	+	+	+	−	II
SA H21	+	+	+	+	+	+	+	+	+	−	IVb
SA H22	+	+	+	+	+	+	+	+	+	−	II
SA H23	+	+	+	+	+	+	+	+	+	−	IVa
SA H24	+	+	+	+	+	+	+	+	+	−	II
SA H25	+	+	+	+	+	+	+	+	+	−	II
SA H26	+	+	+	+	+	+	+	+	+	−	I
SA H27	+	+	+	+	+	+	+	+	+	−	IVd
SA H28	+	+	+	+	+	+	+	+	+	−	IVa
SA H29	+	+	+	+	+	+	+	+	+	−	IVd
SA H30	+	+	+	+	+	+	+	+	+	+	II
SA H31	+	+	+	+	+	+	+	+	+	−	I
SA H32	+	+	+	+	+	+	+	+	+	−	NT
SA H33	+	+	+	+	+	+	+	+	+	−	II
SA H34	+	+	+	+	+	+	+	+	+	+	II
SA H35	+	+	+	+	+	+	+	+	+	−	NT
ATCC700698	+	+	+	+	+	+	+	+	+	−	II
ATCC25923	+	+	+	+	+	+	+	+	−	−	−

^a^MF represents mannitol production, Blood lysis represents hemolysis on blood agar, Biofilm represents biofilm production, *mecA* represents gene encode for penicillin-binding protein 2A (PBP2A); *pvl* represents gene encode for Panton-Valentine leukocidin toxin. + and – represent present and absent; NT denotes *SCCmec* cassette non-typeable

The *coa* gene amplification produced amplicons size in the range of 550 to 800 bp, the amplicons of 700 bp showed the highest frequency (33.33%) followed by amplicon sizes of 550 bp (26.66%), 650 bp (13.33%), 740 bp (0.66%), 660 bp (0.66%), 800 bp (0.66%) and 600 bp (0.66%) (Supplementary Fig. 5). The result of the *coa* gene PCR–RFLP is summarized in supplementary Table 3 and supplementary Fig. 6. To get more insight into the similarity and difference of complex RFLP banding pattern, presence/absence heat-map and dendrogram was generated. Visualisation of banding patterns revealed six distinct clusters, namely A-F with calculated prevalence of 6.6, 13.3, 13.3, 20, 33.3 and 13.3%, respectively. (Supplementary Fig. 7). Typing of *coa* gene and *HaeIII* RFLP, as well as DIs, were presented in supplementary Table 3.

Typing of the *spa* gene revealed 10 amplicons, ranging in size from 355 to 560 bp (Supplementary Fig. 8). The DI of *spa*-PCR typing was 0.9429. Analysis of the *spa* gene revealed twelve known *spa* types. SA G11 and SA H29 strains possessing *SCCmec*-IV gene (CA-MRSA) and both were in the same t008 *spa*-type. SA H16 and SA H19 strains clustered together into t062 *spa*-type and both were found resistant to erythromycin, also classified into HA-MRSA group (Supplementary Table 4). The phylogenetic tree based on *spa* sequences revealed four distinct clusters, designated as A, B, C, and D with a prevalence of 33.33%, 46.66%, 13.33%, and 6.66% respectively (Supplementary Fig. 9).

Although, all the selected strains did not produce biofilm each of them harbors genes for intracellular adhesion (*icaADBC*) and regulation (*icaR*). The presence of *fnbA* and *fnbB* genes were detected in 73.3% and 66.6% strains, respectively. Genes also associated with biofilm-forming ability viz. *cna*, *clfA*, *clfB*, and *ebps* were found present in 53.33%, 80%, 73.3%, and 86.6% strains, respectively. Agarose gel electrophoresis pictures of biofilm-associated genes are presented in supplementary figs. 10–20.

### Cluster analysis based on phenotypic and genotypic data

Although, the individual test, for example, performed AST, catalase, coagulase, DNase, citrate utilization, urease production, mannitol fermentation, blood lysis, and biofilm production assays has the advantage of being cost-effective, but often cannot differentiate among the strains. PCR-based detection of *mecA* gene and genes responsible for PIA (*icaADBC* and *icaR*) could not differentiate the clinical isolates in the present study. PCR-based detection of *pvl* gene and genes encoding for MSCRAMMs (*fnaA*, *fnaB*, *clfA*, *clfB*, *cna*, and *ebps*) also showed poor DP, while *SCCmec*, *coa*-HaeIII RFLP and *spa* typing revealed moderate DP. To avoid the misleading conclusion, the data from all applied methods were coupled to perform cluster and PCA analysis. The generated dendrogram suggested that strains collected from the same geographical region shared the same cluster F. Except for cluster E in which strains SA G5 and SA H29 from different geographical locations clustered together (Fig. 1b). The PCA analysis also revealed that SA G5 and SA H29 shared less variance than the other strains (Fig. 1c).

### General features of sequenced genomes

Ion-Torrent PGM sequencing of SA G5 and SA H29 genomes generated reads range of 18.55 and 25.98 million bases (Mb) per sample covering more than 98% of the reference genome (ASM1342v1) with an average depth of 152.4X. SPAdes assembled sequences were further analyzed by QUAST producing genomes with 43 and 47 contigs and N50 ranging from 85 and 185 kb after filtering contigs size < 200 bp. Mauve contigs ordering and MeDuSa scaffolder produced SA G5 and SA H29 final genomes sequence lengths of 2,760,385 and 2,834,624 bp respectively. The total GC contents were 32.77 and 32.65%. The SA G5 and SA H29 genomes contain 2689 and 2843 coding sequences (CDS) with 9 and 6 rRNAs, and 59 and 51 tRNAs, respectively. The summarized genomic features were shown in Table 2.

### Comparative genome analysis

OrthoANIu identity found ANI of 97.20% among the SA G5 and SA H29 genome sequences. Similarly, dDDH was calculated using the GGDC tool revealed that the probability of DDH (DNA-DNA hybridization) is 84.5% with a genetic distance of 0.0308, suggesting that SA G5 and SA H29 occupy identical taxonomic status which is also supported by the different genotypic data.

**Fig. 2:**
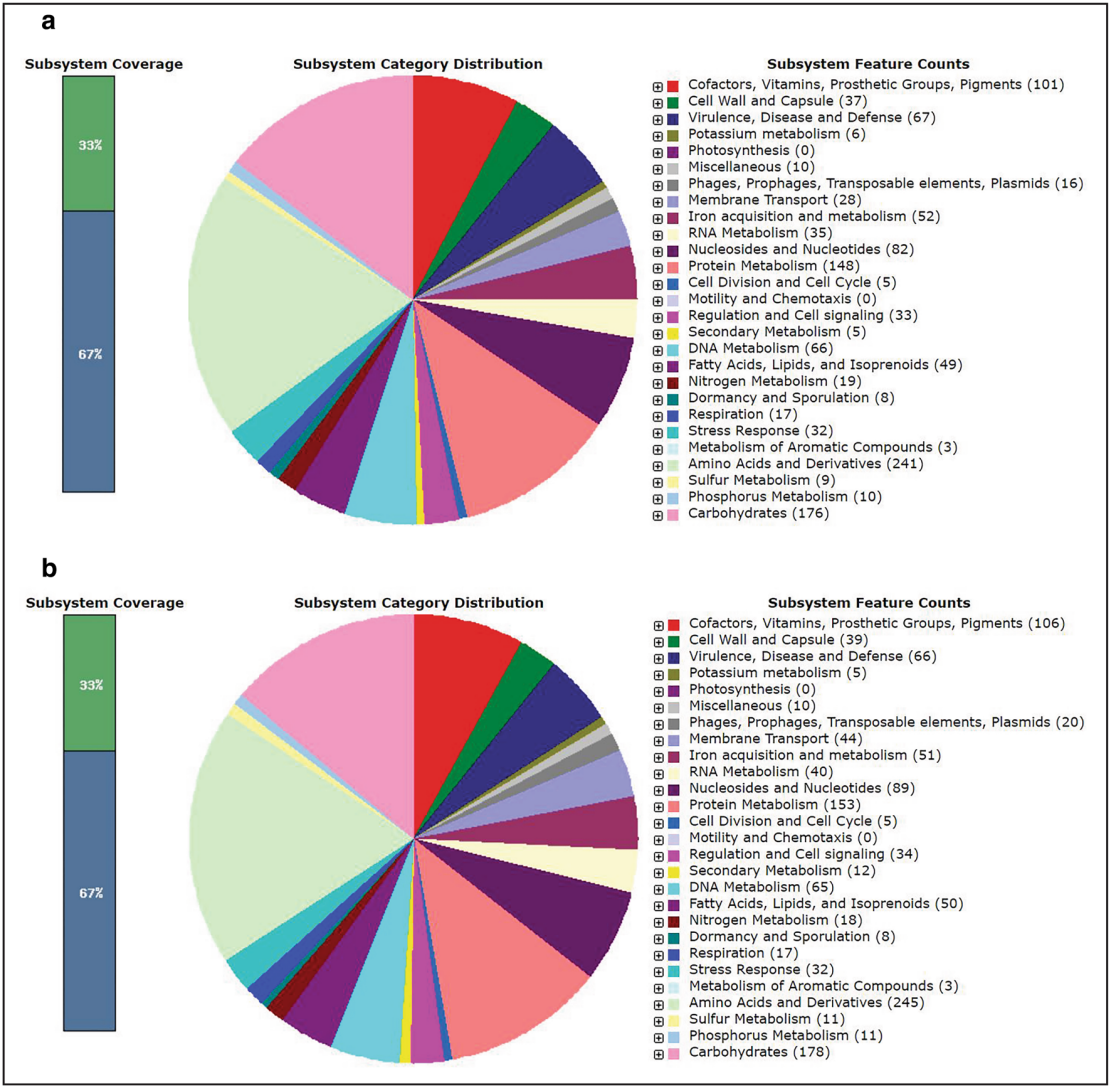
Subsystem category distribution of **(a)** SA G5 and **(b)** SA H29 genomes. The genomes of SA G5 and SA H29 annotated using the RAST Server classified subsystems into 270 and 276 respectively. The green part in the bar-chart at the leftmost position corresponds to the percentage of proteins included and the pie-chart in the right panel demonstrates the subsystem category distribution

**Fig. 3:**
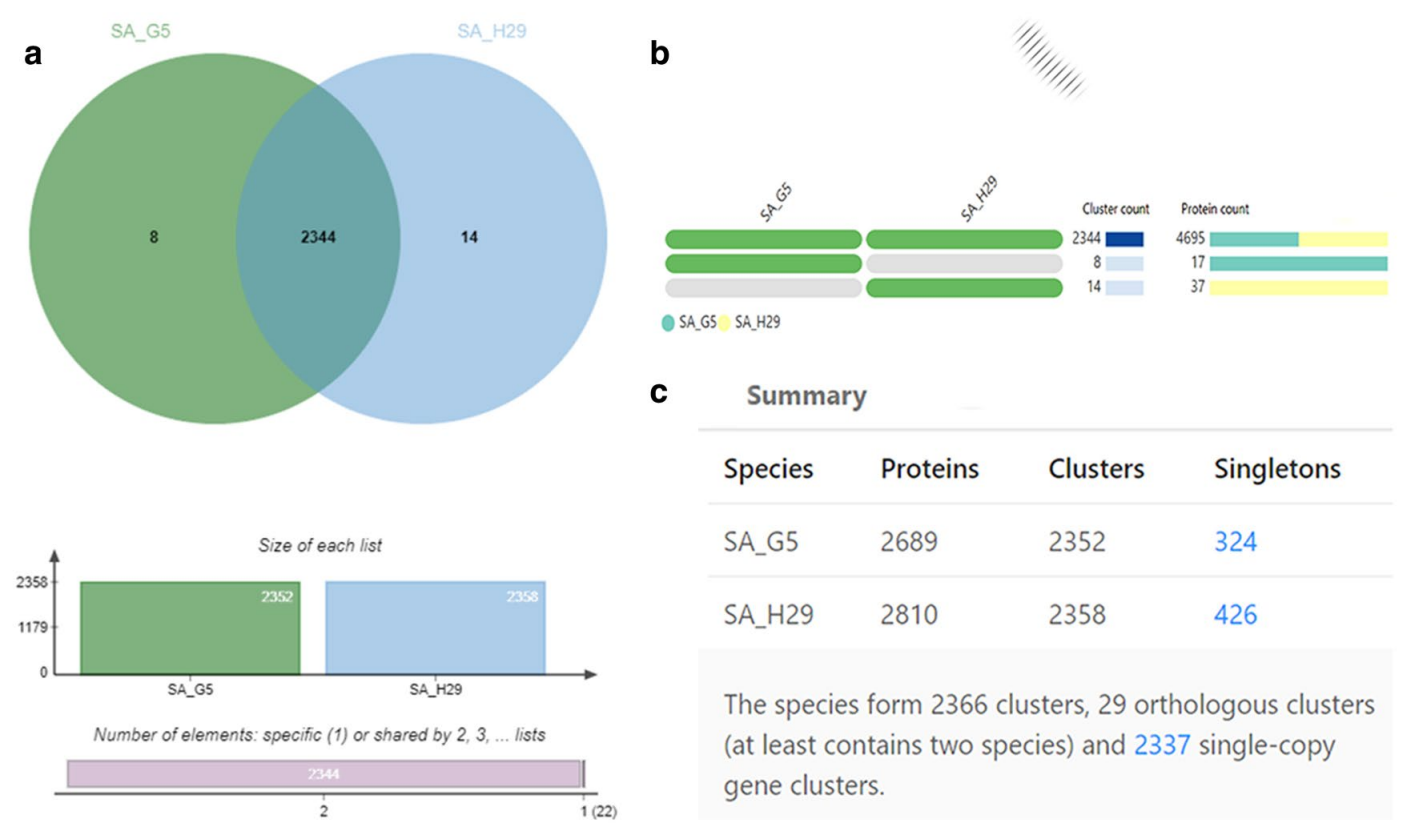
Comprehensive comparative protein analysis of SA G5 and SA H29. **a** Venn diagram showing the distribution of shared orthologous clusters (core-genome) among the genomes. **b** Occurrence pattern of shared orthologous groups among SA G5 and SA H29. The pattern to the left indicates SA G5 and SA H29 genomes are in the clusters, the number of clusters shared between genomes (cluster count), and the number of protein members in the shared clusters (protein count). **c** Showing the numbers of proteins, cluster genes, and singletons with respect to species

**Fig. 4:**
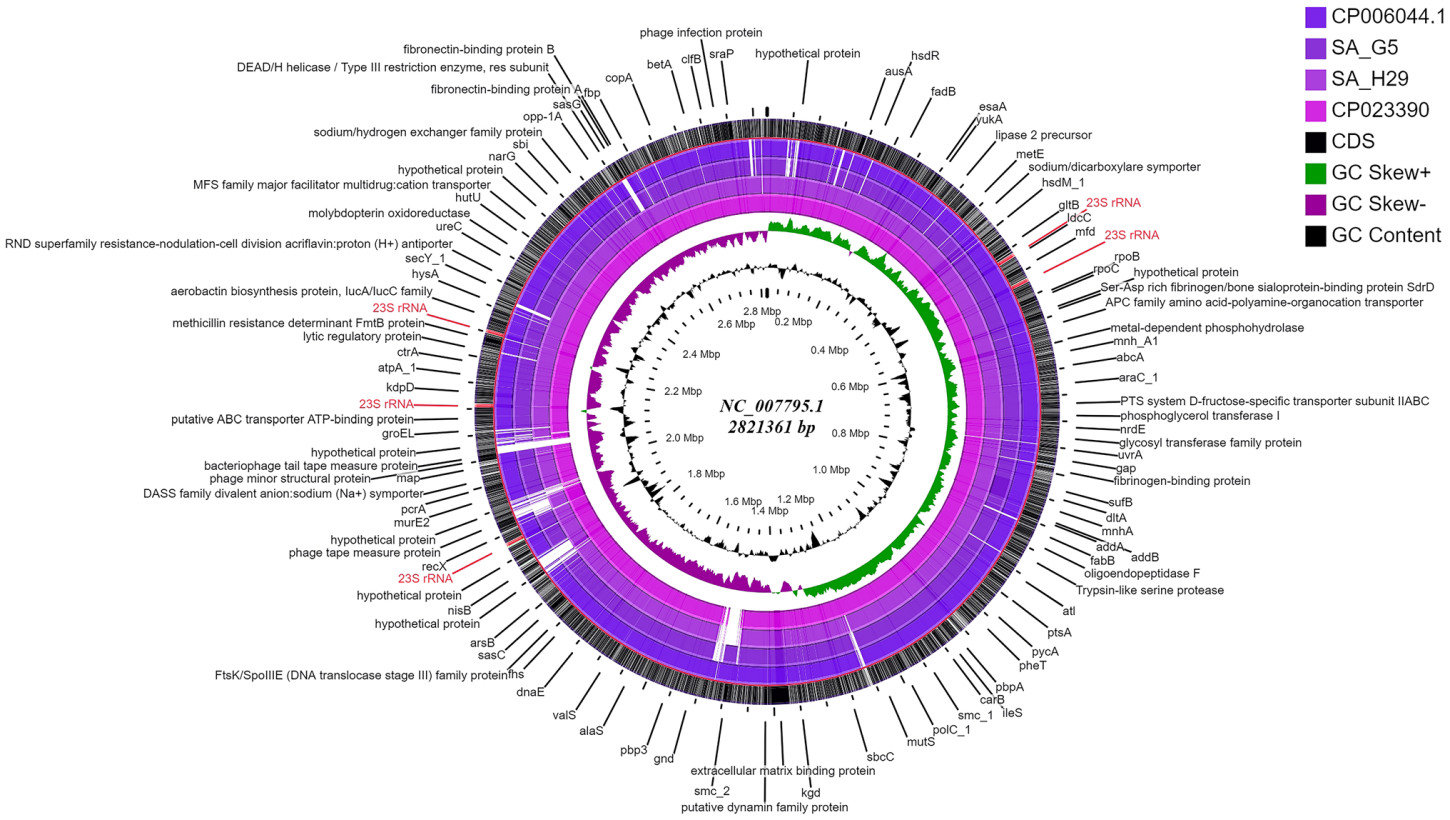
Circular genome comparison map showing homologous chromosome of *S. aureus* genomes against *S. aureus subsp. aureus NCTC 8325* (NC_007795.1) genome using CGviewer. The inner scales designate the coordinates in mega-base pairs (Mbp). White spaces indicate regions with no identity to the reference genome and the genome features were indicated by the outermost (black) ring

According to SEED subsystems, SA H29 genome contains amino acids and derivatives encoding genes (245 CDSs), virulence, disease and defense genes (67 CDSs), carbohydrate utilization genes (178 CDSs) and genes derived from phages, prophages, transposable elements and plasmid (20 CDSs) as presented in the Fig. 2a, b. In addition, the relationship between SA G5 and SA H29 genomes were analyzed using web platform OrthoVenn2, which identified 2366 gene clusters (pan-genome), of which 2344 orthologous clusters were shared between SA G5 and SA H29, and 2337 were single-copy gene clusters (Fig. 3c). These two strains shared 2344 gene clusters (core-genome) comprising 4695 proteins (Fig. 3a, b). SA H29 genome possesses the highest singleton genes covering 37 numbers of proteins that are responsible for virulence, resistance, mobile genetic elements, and lanthionine biosynthesis. Whole-genome circular comparative map of two genomes (SA G5 and SA H29) and their close genomes against *Staphylococcus aureus subsp. aureus NCTC 8325* (NC_007795.1) was generated using CGView server based on BLAST sequence similarities (Fig. 4). Each genome was represented by a different color and the darker areas in the circular genome showed a 100% sequence similarity with the reference genome, whereas the lighter areas showed a 70% sequence similarity.

Epidemiological characterizations of *SCCmec* type, *spa* type, and multilocus sequence type (MLST) are presented in Table 2. In silico analysis of ARGs using CARD database revealed 18 genes related to antibiotic resistance and among them, 16 genes were shared which are responsible for the resistance against methicillin (*mecA*), beta-lactams (*blaZ*), fluoroquinolone (*norA, gyrA,* and *gyrB*), tetracycline (*tet-38*), glycylcycline (*mepA*), multidrug and toxic compound (*mepR*), fluoroquinolone and acridine dye (*arlS* and *arlR),* tetracycline, penam, cephalosporin, glycylcycline, rifamycin, phenicol, triclosan and fluoroquinolone (*mgrA*), daptomycin (*clsA*), diaminopyrimidine (*dfrC*), nitroimidazole (*msbA*), rifamycin (*rpoB32*) and defensin (*mprF*). In addition, genes that provide resistance against macrolide, lincosamide, streptogramin (e*mrA*) were detected in the SA H29 genome. *In-silico* identification of ARGs and the results of *in-vitro* AST (for erythromycin, vancomycin, and beta-lactam antibiotics) are in good agreement.

The VFGs were predicted against the VFDB in the PATRIC annotation system. Genomic comparison of the two strains identified 78 genes that encode virulence factors of which 57 are common. These virulence factors are responsible for adherence, toxins production, antiphagocytosis, immune evasion, exoenzyme activity, iron uptake, and secretion system. Genomes annotation revealed common genes encoding MSCRAMMs *i.e*. adhesion (*sdrC, sdrD*, and *sdrE*) elastin binding protein (*ebps*), polysaccharide intercellular adhesion proteins (*icaA*, *icaB*, *icaC*, *icaD*, and *icaR*), clumping factor (*clfB*) and cell wall anchored protein (*sasH*). The genome of SA H29 isolate harbored all these genes extended with additional genes encoding clumping factor A and B (*clfA* and *clfB*), fibronectin-binding protein A (*fnbA*), serine-aspartate repeat-containing proteins (*sdrC* and *sdrD*), and fibronectin-binding protein (*fnbp*), while the genome of SA G5 contained additional genes responsible for collagen adhesion (*cna*), and extracellular adherence protein (*eap*/*map*).

Several hemolysin toxin genes such as alpha (*hla*), beta (*hlb*), delta (*hld*) and gamma A, B, and C (*hlgA, hlgC*, and *hlgB*) were identified in both the strains. Bicomponent leukotoxins, leukocidin *lukE*, and *lukD* genes were identified in the SA H29 genome. In the sequenced genomes 10 Staphylococcal enterotoxins (SEs) types were detected. The most prevalent enterotoxin genes, *sea*, and *seb* were presented in SA H29. The genome of SA G5 possessed 8 enterotoxin gene types such as *sec*, *sec3*, *seg*, sei, *sel*, *sem, seo,* and *seu*. Toxin genes involved in immune evasions such as IgG-binding proteins (*sbi* and *spa*), staphylococcal complement inhibitor (*scn*), and staphylokinase (*sak*) were present in both genomes. Chemotaxis-inhibiting protein (CHIPS) encoded by *chp* was identified in the SA G5 genome. The antiphagocytosis capsular stereotype encoding genes such as *cap5C, cap5D, cap5F, cap5G, cap5O, cap5P, cap8M*, and *cap8N* were present in both genomes, however, *cap5E* gene was detected in SA H29 genome. Moreover, capsular stereotype 8 genes (*cap8E* and *cap8H*) were detected in SA G5 genomes. *S. aureus* secret ESAT-6-like proteins consist of eight genes cluster namely *esxA, esxB, esaA, esaB, esaC, essA, essB,* and *essC*. These eight secretary system genes were identified in both genomes. Several exoenzyme encoding genes namely staphylocoagulase (*coa*), catalase (*katA*), hyaluronidase (*hysA*), von Willebrand factor binding protein (*vwb*), zinc metalloproteinase aureolysin (*aur*), V8 protease/glutamyl endopeptidase (*sspA*), staphopain B (*sspB*), staphostatin B (*sspC*) and lipase (*geh*) were present in both genomes. Genes involved in iron uptake mechanism such as *isdA, isdB, isdC, isdD, isdE, isdF, srtB,* and *isdG* that encodes for cell surface protein, cell surface receptor, cell wall anchor protein, heme-transporter component, high-affinity heme-uptake system protein, heme–iron transport system permease protein, sortase B and heme-degrading monooxygenase/staphylobilin-producing respectively, were identified in both genomes.

The putative plasmids were identified in nonaligned contigs that displayed an unexpected high coverage level after the genome assemblies. Two putative plasmids, p1H29 and p2H29 of 17,165 bp and 9020 bp lengths, respectively were identified in nonaligned contigs (scaffolds 2 and 3) of SA H29 genome, and these two plasmids constitute *rep20* and *rep7C* type genes. Plasmid p1H29 has and showed 44.20% and 57.34% sequence coverage with plasmids pBU108b (KF831356.1) and pPS00089.1A.1 (NZ_CP022911.1) respectively. Plasmid p2H29 has a length of and showed 23.42% and 23.45% sequence coverage with plasmid: II (LT671860.1), and UP_1395 plasmid (NZ_CP047821.1) respectively. Identified plasmid p1H29 carried genes encoding cadmium resistance (CadD) and transportation (CadX) proteins, and enterotoxins (EntA, EntD and EntG). Identified plasmid, p2H29 has *blaZ* (beta-lactamase), *blaR,* and *mecI* genes that conferred resistance to penicillin. The identified plasmids of *S. aureus* encode no transfer factors; thus, these plasmids may transfer via bacteriophage generalized transduction (McCarthy and Lindsay 2012).

**Fig. 5:**
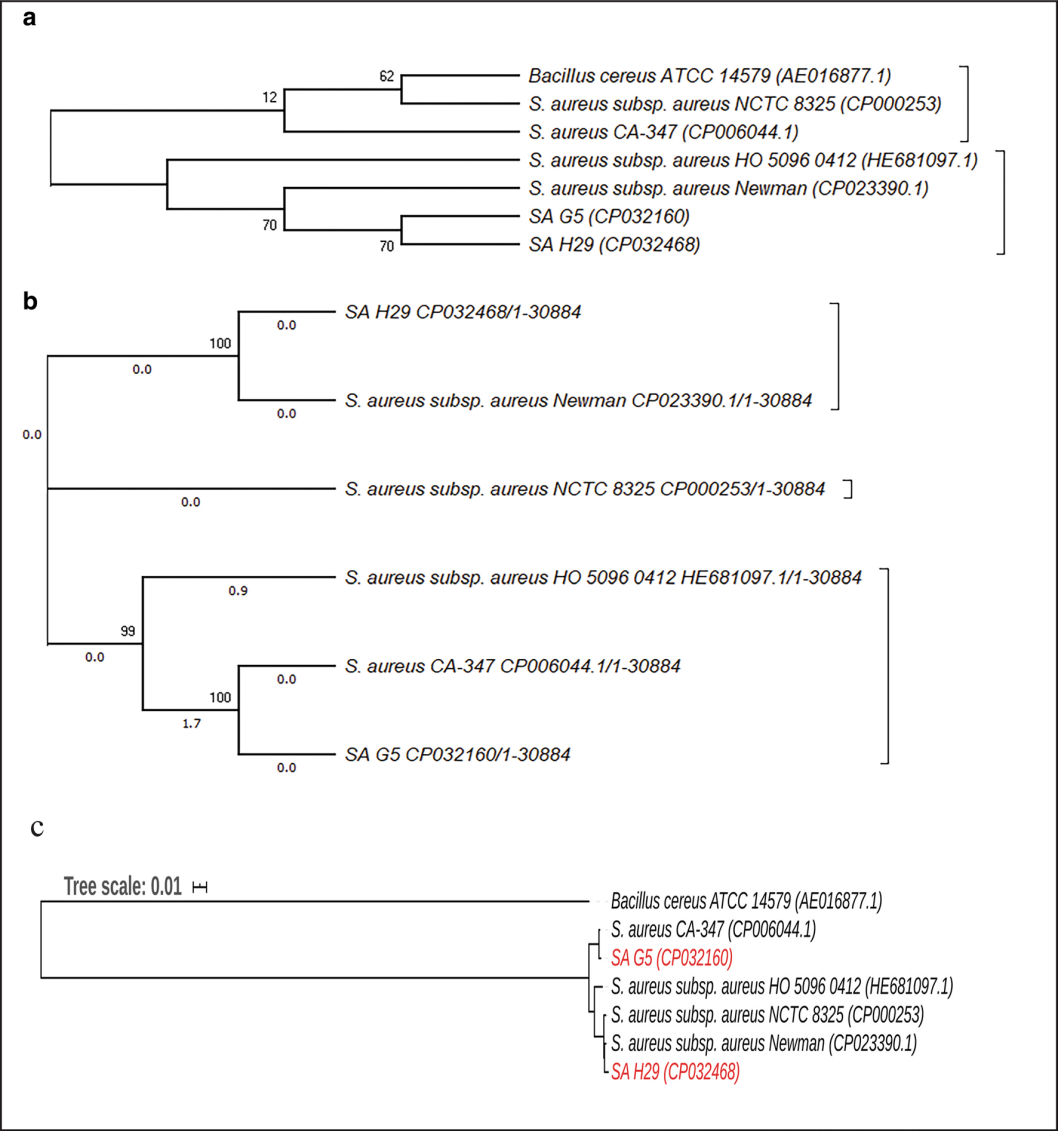
Comparative phylogenetic analysis of SA G5 and SA H29 strains with their closely related *S. aureus* strains. **a** Neighbor-joining tree prepared from the 16S rRNA gene sequences of SA G5, SA H29, and other related strains. *Bacillus cereus* ATCC 14,579 was used as an out-group and branch values are calculated from 500 bootstrap replicates. **b** Maximum likelihood (ML) tree based on all shared proteins (core) obtained from the *S. aureus* genome sequences, where *B. cereus* ATCC14579 was used as an out-group. The ML tree was prepared with the help of RAxML program. **c** Phylogeny tree based on the concatenated alignment of the high-quality SNPs using CSI phylogeny tool

A comparative phylogenetic relationship was analyzed using three different gene sequences (viz., 16S rRNA genes, SNPs, and genes encoding core protein) to compare at the strain level. The phylogenetic tree generated using 16S rRNA gene sequences showed that SA G5 and SA H29 strains are highly similar and grouped them in the same group (Fig. 5b), however, the phylogenetic tree prepared from the SNP sequences and protein-coding gene sequences grouped them differently suggesting that 16S rRNA gene sequences are not sufficient to discriminate them on the strain level, possibly due to the low resolution of their evolutionary relationships (Konstantinidis and Tiedje 2007). It is interesting that both these methods and ANI values support each other and can be useful in distinguishing the genomes even in the strain level (Fig. 5b, c).

## Discussion

In the present study thirty-five European *S. aureus* clinical isolates were characterized by several different typing methods. The data generated from typing results along with virulence genes detection were evaluated through cluster analysis. Antibiotic susceptibility test and detection of *mecA* gene result showed 33 (94.28%) strains were MRSA while 2 (5.72%) strains were MSSA. Such a validation process was reported by other researchers (Skov et al. 2006; Kumurya 2015). Among the tested strains, 26 strains (71.43%) were found to be resistant to erythromycin. Prevalence of erythromycin-resistant strains collected from Hungary representing 90.47% in a good agreement with previous data (Szabó et al. 2009). It was also reported that the frequency of erythromycin-resistance during 2010–2015 was 72% in Germany (Walter et al. 2017) and 60% in Greece (Stefanaki et al. 2017). However, in the present study, no vancomycin-resistant strains were found which is supported by the observations of previous studies (Szabó et al. 2009; Chaudhari et al. 2014). Vancomycin kills Gram-positive bacteria by interfering with peptidoglycan synthesis and peptidoglycan assembly (McGuinness et al. 2017). The vancomycin susceptibility can be helpful in the treatment of MRSA infections. The resistance of *S. aureus* to vancomycin is governed by many factors. Previous studies have shown that point mutation in different regulatory loci associated with cell wall metabolism including two-component regulatory systems (guanylate kinase gene (*gmk*); *walRK*, *graSR,* and *vraSR*) resulted in vancomycin-resistant *S. aureus* strains (McAleese et al. 2006; Howden et al. 2008; Cui et al. 2009, 2010; Cameron et al. 2012; Shekarabi et al. 2017). In addition to cell wall thickening, altered surface protein profile, enhanced capsule, and *agr* gene dysfunction are also found to be the cause of the generation of vancomycin-resistant *S. aureus*. The presence of the *vanA* operon is also found to confer vancomycin-resistant (Périchon and Courvalin 2009; McGuinness et al. 2017). Also, it was observed that vancomycin-susceptibility in MRSA due to the activation of the WalRK two-component regulatory system (Cameron et al. 2016). The stains of the present study may neither have these mutations or cell wall-related modifications nor do they carry the genes required for vancomycin resistance and thus showed susceptible phenotype. However, a detailed study will be required to confirm the presence or absence of these factors.

Some of the MRSA strains produce PVL, encoded by two genes, *lukS-PV*, and *lukF-PV* which have been shown to play a role in the pathogenicity of *S. aureus* by provoking necrosis, accelerating apoptosis and destruction of polymorphonuclear and mononuclear-cells, thereby contributing to morbidity and mortality (Lina et al. 1999). It was reported that the low prevalence of *pvl* has found 5% and 4.9% in MRSA strains isolated from France and the UK respectively (Holmes et al. 2005). In the present study, 24.24% (8/33) strains were found positive to the *pvl* gene. Among these strains, three carried *SCCmec* type IV and one classified as *SCCmec* type V, however, four strains carried *SCCmec* II (Table 2). According to previous reports, MRSA strains belong to *SCCmec* types I, II, and III are dominant among the HA-MRSA, while *SCCmec* types IV and V characteristic of CA-MRSA (Monecke et al. 2011; Chua et al. 2014). In the present study, we found that *SCCmec* type II prevalence (12.12%) was higher in comparison with other *SCCmec* types. It was also reported that *SCCmec* type II usually presents in multidrug-resistant MRSA strains (Ito et al. 2001; Hiramatsu et al. 2001) and were dominant outside European countries (Kilic et al. 2006; Makgotlho et al. 2009; de Oliveira et al. 2015). Our data related to *SCCmec* type IV showed a higher prevalence (27.6%). The reason behind this observation is probably due to the easy acquisition of short size *SCCmec* type IV cassette (Robinson and Enright 2004). Even though the representation of non-typeable *SCCmec* in our case complies with the previous finding (Makgotlho et al. 2009), a few non-typeable *SCCmec* can be reduced by applying the new *SCCmec* cassette detection (Kaya et al. 2018). Some of the MRSA strains harbored *SCCmec* IVa showed signs of *pvl* gene negative (Table 2), which is similar to the finding, reported earlier (Moroney et al. 2007). Our data related to *pvl* gene detection revealed that MRSA strains harboring *SCCmec* IVb and *SCCmec* IVd were found negative. Our finding also supports the idea that the harboring bacteriophage *pvl* gene by MRSA strains may not be a promising marker for CA-MRSA (Rossney et al. 2007). This conclusion is supported by other studies about *SCCmec* typing for the classification of HA-MRSA and CA-MRSA (Monecke et al. 2011; Chua et al. 2014). Taken together, our finding suggested that the *SCCmec* typing method is more informative in problem-solving approaches (control and prevent infections caused by MRSA strains) for the clinicians and epidemiologists.

*S. aureus* secretes the coagulase enzyme, a polypeptide that helps promote the clotting of plasma or blood (Cheng et al. 2010). The *coa* gene shows heterogenicity in the 81 base-pair long tandem repeats region differing in number and location of restriction sites among the *S. aureus* isolates (Goh et al. 1992; Schwarzkopf and Karch 1994). The assay based on PCR amplification of the *coa* gene followed by RFLP was used to differentiate among the geographically diverse MRSA strains. This technique is simple, rapid, specific, inexpensive, and reproducible; allowing early recognition of an epidemic strain in a hospital setting (Martineau et al. 1996; Hookey et al. 1998; Shopsin et al. 2000; Mahmoudi et al. 2017). In this study, *coa*-PCR typing yielded seven different amplicons in size range from 550 to 800 bp. Among 15 *S. aureus* strains, the highest occurrence size is 700 bp (33.33%) as shown in Table 4, however, it was earlier reported that 600 bp amplicon is the most predominant (Mahmoudi et al. 2017). Discrimination of *coa* gene-specific amplicon pattern was further improved by HaeIII restriction enzyme digestion, which yielded 11 types of patterns with DI of 0.9619. Our data were in good agreement with the previous result in which DI was improved by digestion (Janwithayanuchit et al. 2006). *S. aureus* produces protein A, an antiphagocytic protein that is coded by the *spa* gene (Shakeri et al. 2010). The analysis of *spa*-sequence revealed high diversity, however, two strains belonged to *spa*-type t008, and another two were classified to *spa*-type t062 (Supplementary Table 3). In a previous study conducted on German, *S. aureus* isolates reported that t003 and t008 were predominant *spa* types (Strommenger et al. 2008). Also, a recently published article stated that t008 was the most prevalent *spa* type in Europe and America (Asadollahi et al. 2018). The *spa*-PCR typing method produced eleven different genotypes with variable amplicons size ranges from 335 to 560 bp and revealed 0.9429 DI, which provides similar DI with *coa*-HaeIII RFLP method which is supported by a previous study (Omar et al. 2014). This study suggested that *spa*-typing has performed better than other molecular typing methods and showed better DP. This typing method is useful for studying the genetic diversity of *S. aureus* for the epidemiological tracking of the source of infections (Koreen et al. 2004) and offering several advantages in comparison with alternatives methods, such as a publicly available comprehensive and curated database for analyzing *spa* sequence with standard nomenclature (Strommenger et al. 2008).

In a good agreement with previous data, we found that not all *ica*-positive isolates produce biofilms (Møretrø et al. 2003; Fitzpatrick et al. 2005), however, the clinical origin of the used *S. aureus* isolates was supported by the presence of *icaADBC* and *icaR*, indicating the ability to discriminate between the normal floras and virulence strains representatives (Galdbart et al. 2000). In this study, we observed that 66% of the isolates harbor two *fnb* genes almost similar to the results reported by Peacock et al. (2000) for European *S. aureus* strains. However, the presence of *fnb* genes in an isolate does not guarantee the biofilm-forming ability of the isolate.

In this study, the *cna* gene was found to be present in 53.3% isolates. Earlier, the prevalence of the *cna* gene was reported in a range from 22 to 56.5% (Peacock et al. 1999; Rohde et al. 2007; Zmantar et al. 2008), The *clfA*, *clfB,* and *epbs* genes play an initial role for biofilm development (Ghasemian et al. 2015), however, our data showed that the presence or absence of these genes does not represent a clear discriminative marker for differentiating strains in terms of biofilm-forming ability.

The grouping of the isolates based on the results of *coa-Hae*III*-*RFLP and *spa* gene sequences produced different groups with different strains. Therefore, the results of all the biochemical and genotypic tests were used to prepare a dendrogram followed by PCA analysis. The dendrogram generated from the combined data of phenotypic and genotypic methods showed that the strain SA G5 and SA H29 collected from different geographical regions clustered together in the same group (Fig. 1b). Similar results were also obtained from the PCA analysis **(**Fig. 1c**).** To gain a better idea about their genetic background and closeness between the two strains, both strains were subjected to whole-genome sequencing and analysis. Comparative genome analysis of these two strains revealed 97.03% OrthoANI identity with a genetic distance of 0.0308, sharing 2344 gene clusters. The genes for virulence, resistance, plasmids, pathogenicity islands, prophage-like elements, and mobile elements are encoded by singleton genes of the SA H29 genome, suggesting that these singleton genes might be inherited through horizontal gene transfer (HGT) events (Lindsay 2014). Besides, the genes (5 genes) encoding lanthionine biosynthesis proteins were also found as singleton genes in the SA H29 genome. The presence of such genes influences the niche adaptation, pathogenesis, and contributes to evolution (Hacker and Carniel 2001). The map revealed a small gap with colorless region against the reference genome, which is due to the change in GC % content, this change in GC % content is due to the acquisition of gene through HGT (Hayek 2013) and the GC skewed regions indicated the regions where HGT occurred.

The 16S rRNA gene sequence-based phylogenetic analysis has been used widely to study the evolutionary relationships of microbes (Janda and Abbott 2002, 2007; Goswami et al. 2017). The phylogenetic tree generated using 16S rRNA gene sequences of the two isolates viz*.*, SA G5, and SA H29 also clustered them in the same group whereas the other publicly available isolates were found in other groups (Fig. 5a) supporting the observations of the dendrogram and PCA analysis. However, the other two phylogenetic trees generated based on the SNP and core protein-coding gene sequences grouped the strain SA G5 and SA H29 in different groups. This indicated that these novel approaches of phylogenetic analysis using core protein-coding gene sequences and SNPs (Fig. 5b, c) are more powerful than the16S rRNA gene sequence-based phylogeny and are generally acceptable to distinguish the genomes even in the genus or species level, higher strain-level resolution. It has been reported that 16S rRNA gene sequence analysis is not powerful enough to discern clearly among the closely related species (Fox et al. 1992). Overall, the results of the present study indicate that whole-genome sequence analysis is more powerful than the individual genotypic test and provide better insight into the taxonomic and genotypic background of the test isolates.

## Conclusion

Methicillin-resistant *S. aureus* is the leading cause of nosocomial and community infections and the emergence of hypervirulent strains and becoming a greater threat to the public. The high-frequency emergence of antibiotic-resistant could be due to the acquisition of resistance determinants such as plasmids, integrons, and transposons through horizontal or vertical gene transfer and partly by improper administration of antibiotics. The phenotypic and genotypic characterizations are important for identifying the risk factors associated with *S. aureus* infections and useful to monitor and control the circulation or transmission of these strains. But a comparative analysis of the pathogens based on the NGS-based genome sequencing technique could extend our understanding of pathogenesis and evolution at the molecular level and has the potential to a breakthrough in diagnosis, treatment, and infection control. In this study, the comparative genomic analysis revealed that niche-specific differences between the *S. aureus* strains in terms of genes and genes clusters that are related to amino acid metabolism, carbohydrate metabolism, cell envelope biogenesis, defense mechanisms, secondary metabolism, and phage-like elements. The difference in the presence of resistance genes (penicillin, methicillin, erythromycin, aminoglycoside, streptothricin acetyltransferase, and cadmium), VFGs (*hlb*, *chp*, *scn*, *ear*, *qsa, sea*, *seb*, *sel*, *seg*, *lukD*, *lukE*, *sasH*, *clfA*, and *eap*), plasmids and phage-related genes between the strains were also observed which may be due to the events like HGT and homologous recombination. Also, the comparative genome analysis provides high resolution to distinguish between the closely related sequenced strains which are indistinguishable by *SCCmec* and *spa* typing. The whole-genome analysis technique is a feasible tool to improve clinical diagnostic investigations of clinical infectious diseases in real-time and provides the goal of improving patient care.

## Electronic supplementary material

Below is the link to the electronic supplementary material.

Supplementary file1 (DOCX 11382 kb)

Supplementary file2 (CSV 3 kb)
