# Supplementary material for: Characterization of methicillin-resistant Staphylococcus aureus through genomics approach
Source: 3 Biotech. 2020 Aug 20;10(9):401. doi: 10.1007/s13205-020-02387-y (PMC7441129; doi:10.1007/s13205-020-02387-y)
Supplement: Supplementary file 1 — Supplementary file1 (DOCX 11382 kb) [file 13205_2020_2387_MOESM1_ESM.docx]

**3 Biotech**

**Title: Characterization of methicillin-resistant *Staphylococcus aureus* through genomics approach**

**Author names:** Romen Singh Naorem^1^, Peter Urban^1,2^, Gunajit Goswami^3^ and Csaba Fekete^1^

**Affiliations and address:** ^1^Department of General and Environmental Microbiology, Institute of Biology, University of Pécs, Pécs 7624, Hungary.

^2^Microbial Biotechnology Research Group, Szentágothai Research Centre, Pécs 7624, Hungary.

^3^Department of Life Sciences, Dibrugarh University, Dibrugarh, Assam 786004, India.

**Corresponding author:** Csaba Fekete

**Email:** [fekete@gamma.ttk.pte.hu](mailto:fekete@gamma.ttk.pte.hu)

**Phone number:** +36-72-503600

**ORCID:** 0000-0001-9430-4643

**
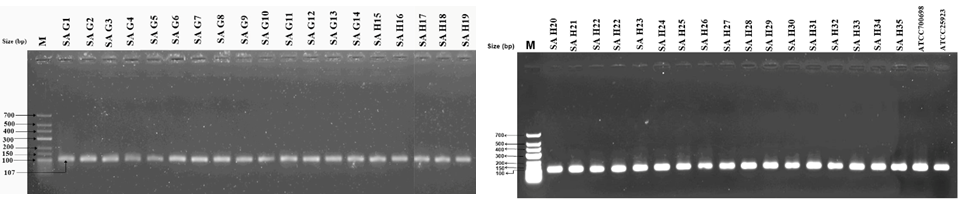
**

**Supplementary Fig. 1** Agarose gel electrophoresis analyses of the PCR amplified products of the species-specific sequence of *S. aureus* clinical isolates. The amplification of a 107 bp product indicates a positive result. Lane M indicates the low-range DNA ladder (Fermentas, USA) and the rest of the lanes indicate the PCR amplified products of a species-specific sequence of *S. aureus* isolates.


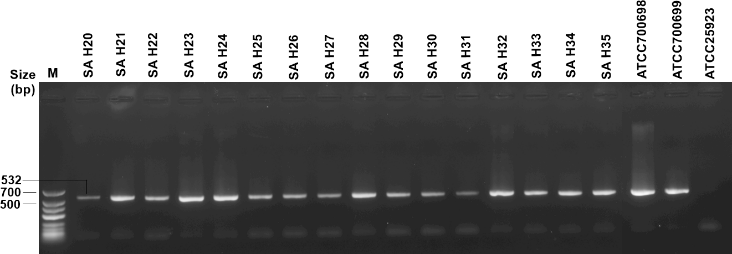

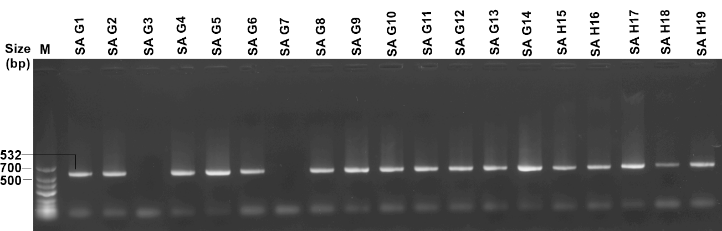


**Supplementary Fig. 2** Agarose gel electrophoresis analyses of the PCR amplified products of *mecA* gene of *S. aureus* clinical isolates. Lane M indicates the low-range DNA ladder (Fermentas, USA) and the rest of the lanes indicate the PCR amplified products of *mecA* gene. A 532 bp amplified product indicates a positive result.


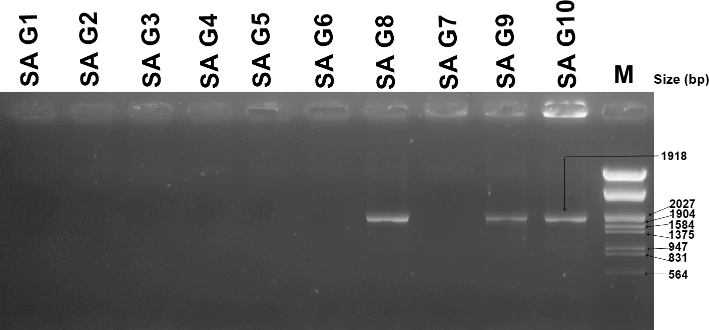

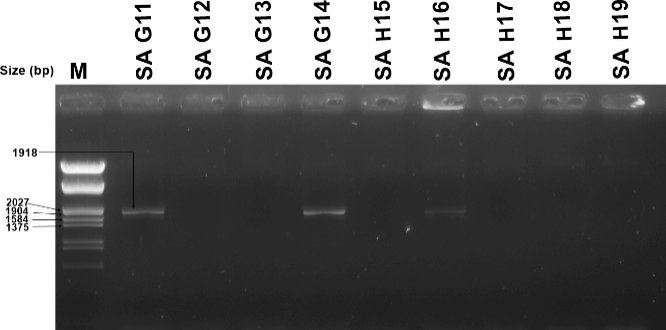

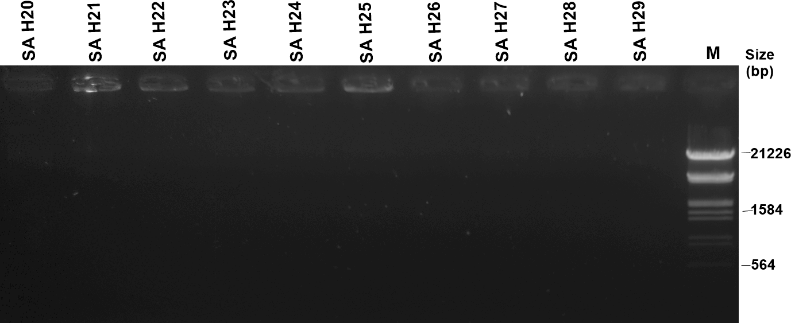

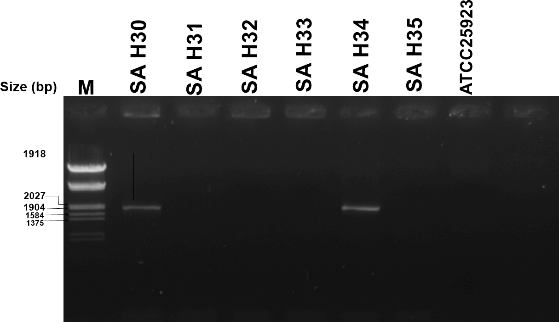


**Supplementary Fig. 3** Agarose gel electrophoresis analyses of the PCR amplified product of *pvl* gene of *S. aureus* clinical isolates. Lane M indicates the Lambda DNA digested with *EcoRI* and *HindIII* Marker 3 (Fermentas, USA). Amplification of 1918 bp indicates a positive result.


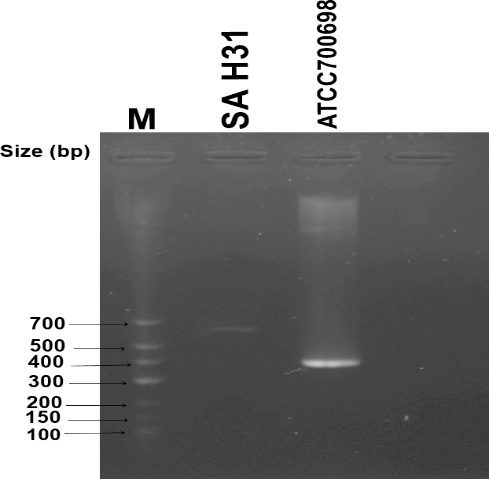

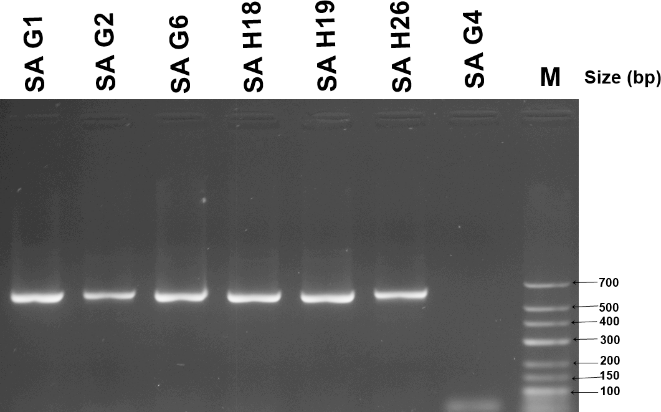


**B.**

**A.**


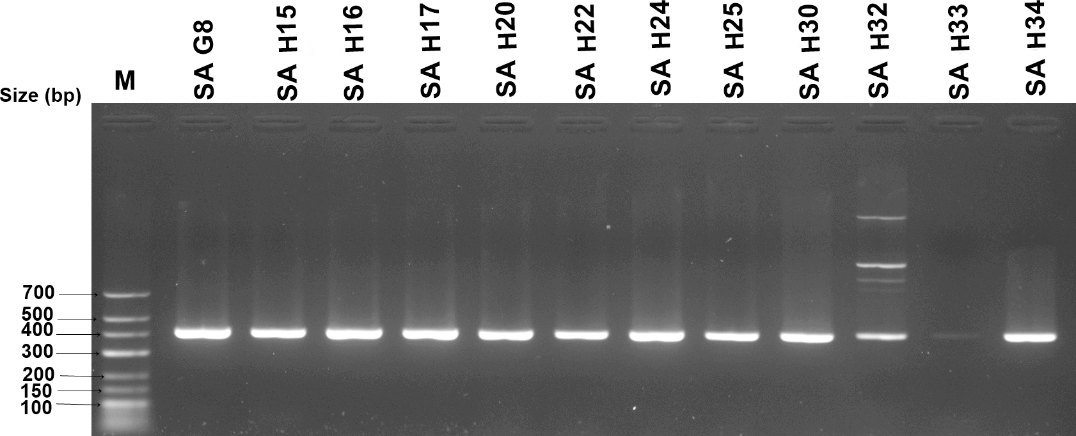


**C.**


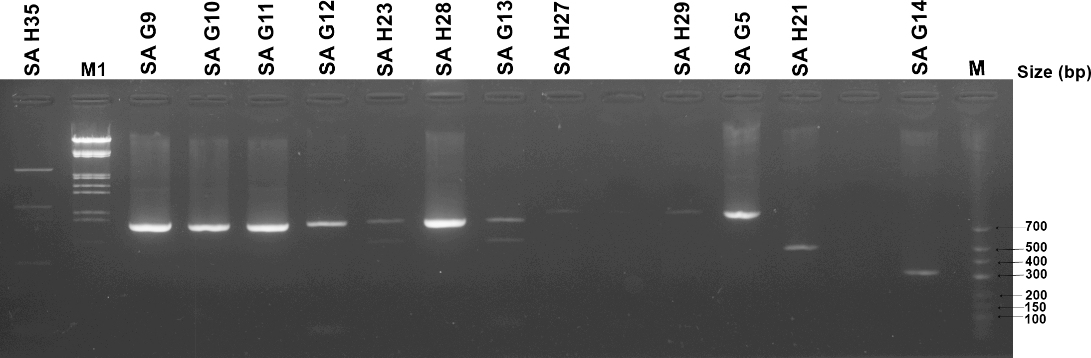


**D.**

**Supplementary Fig. 4** Agarose gel electrophoresis of the PCR amplified products of *SCCmec* gene of *S. aureus* clinical isolates. The PCR amplified product represents *SCCmec* type I are shown in panel (A) and (B); *SCCmec* type II in panel (C); and *SCCmec* type IV and V in panel (D). Lane M indicates the low-range DNA ladder (Fermentas, USA) and Lane M1 indicates the Lambda DNA digested with *EcoRI* and *HindIII* Marker 3 (Fermentas, USA).


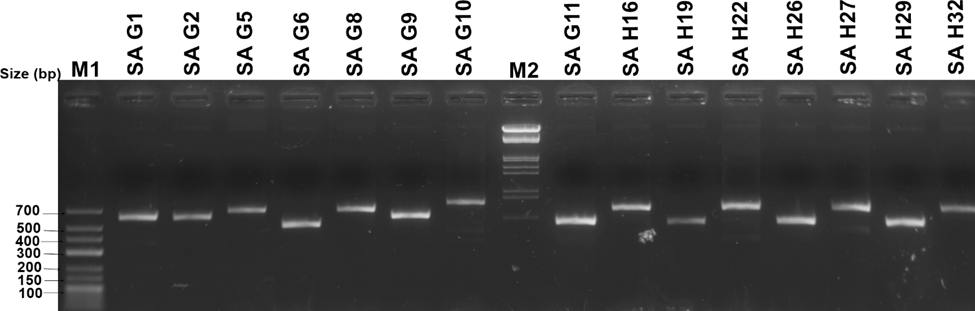


**Supplementary Fig. 5** Polymorphism analysis of the *S. aureus* clinical isolates through Agarose gel electrophoresis of the PCR amplified products of *coa* gene. Lane M1 indicates the low-range DNA ladder (Fermentas, USA) and Lane M2 indicates the Lambda DNA digested with *EcoRI* and *HindIII* Marker 3 (Fermentas, USA).


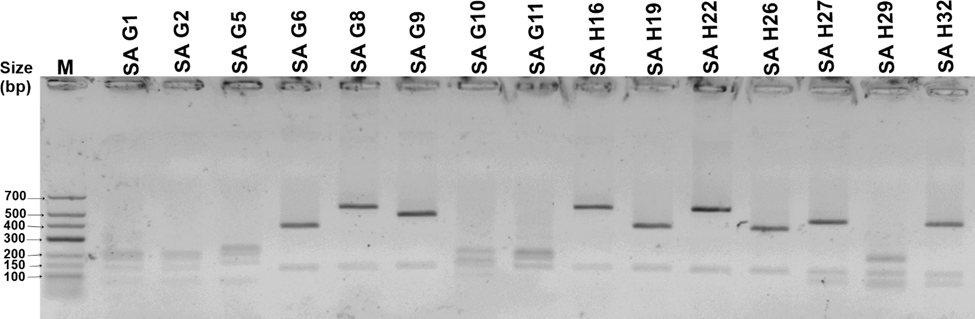


**Supplementary Fig. 6** Agarose gel electrophoresis showing the RFLP pattern of *S. aureus* clinical isolates based on the digestion of *coa* gene with *HaeIII.* Lane M indicates the low-range DNA ladder (Fermentas, USA).


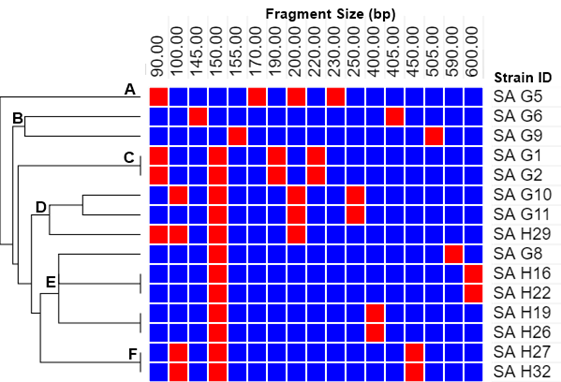


**Supplementary Fig. 7**. Heat-map showing the similarity and difference of complex *coa-HaeIII-*RFLP banding pattern. The top labels indicate the fragment sizes (bp), Strain ID listed at the right part of the panel, and clustering hierarchy demonstrated by the dendrogram at left.


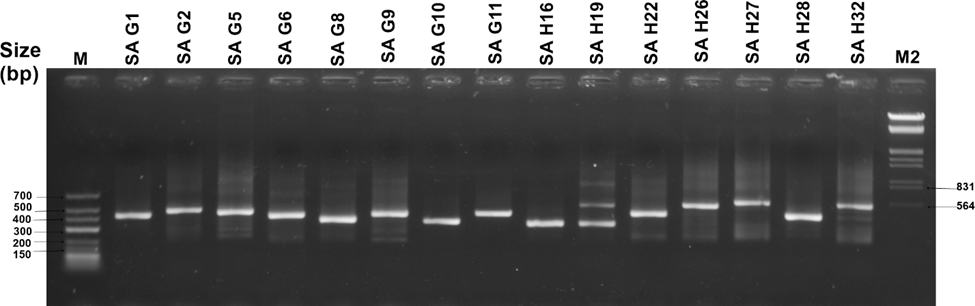


**Supplementary Fig. 8** Agarose gel electrophoresis of the PCR amplified products of *spa* polymorphic gene of *S. aureus* clinical isolates. Lane M indicates the low-range DNA ladder (Fermentas, USA) and Lane M2 indicates the Lambda DNA digested with *EcoRI* and *Hind*III (Fermentas, USA).

**
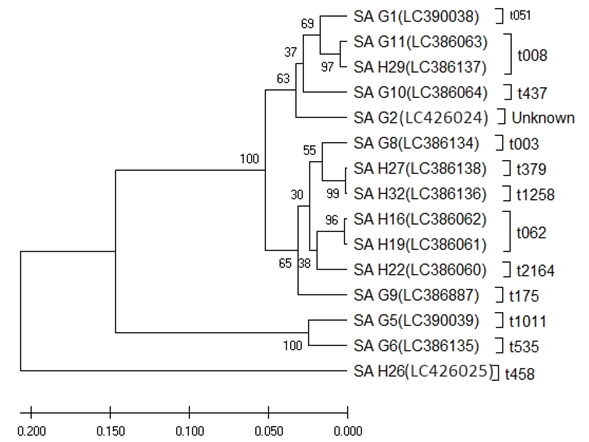
**

**Supplementary Fig. 9** Phylogenetic analysis of *S. aureus* isolates based on *spa* gene sequences using UPGMA method. Strain ID and its *spa*-gene sequence accession number are indicated together with respective *spa*-type. Clusters are denoted by A, B, C, and D. Evolutionary distance is represented on the scalebar. Bootstrap values are presented based on 1000 replicates.


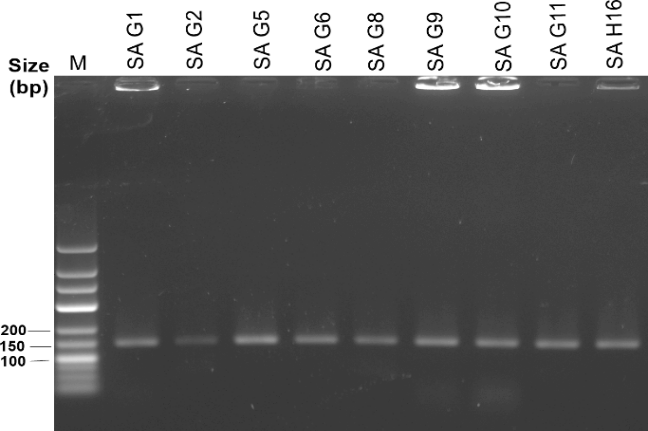

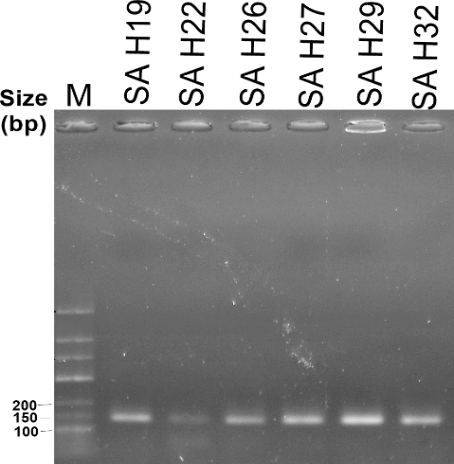


**Supplementary Fig. 10** Agarose gel electrophoresis of the PCR amplified products of *icaA* gene of *S. aureus* clinical isolates. Lane **M** indicates the low-range DNA ladder (Fermentas, USA). A 141 bp amplified product indicates a positive result


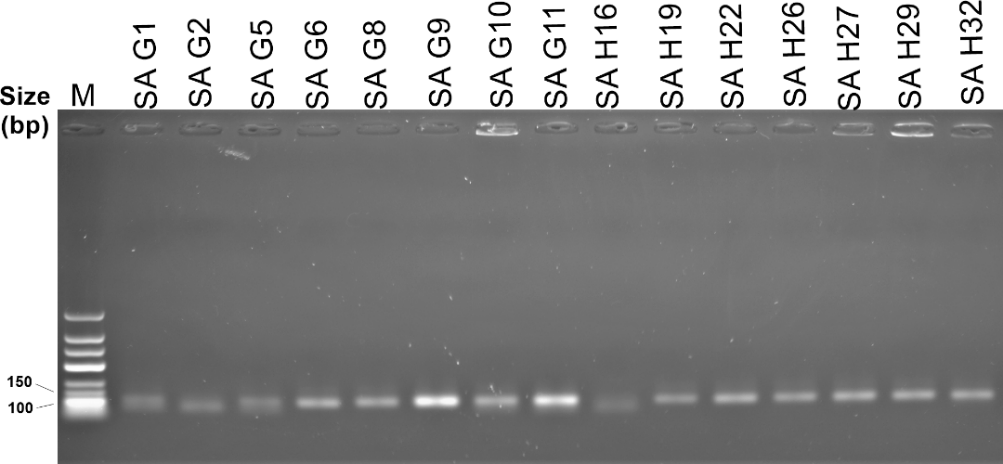


**Supplementary Fig. 11** Agarose gel electrophoresis of the PCR amplified products of *icaB* gene of *S. aureus* clinical isolates. Lane **M** indicates the low-range DNA ladder (Fermentas, USA). A 137 bp amplified product indicates a positive result.


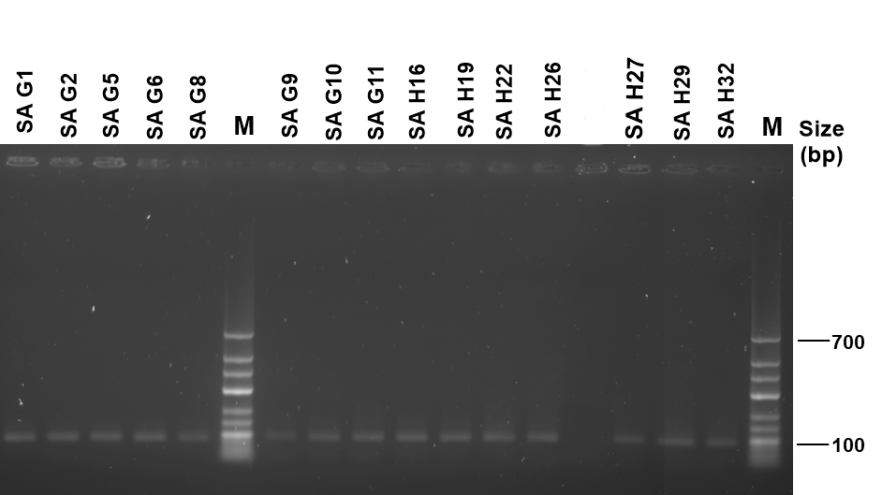


**Supplementary Fig. 12** Agarose gel electrophoresis of the PCR amplified products of *icaC* gene of *S. aureus* clinical isolates. Lane **M** indicates the low-range DNA ladder (Fermentas). A 100 bp amplified product indicates a positive result


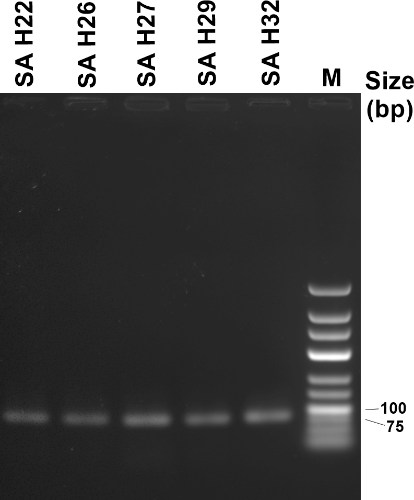

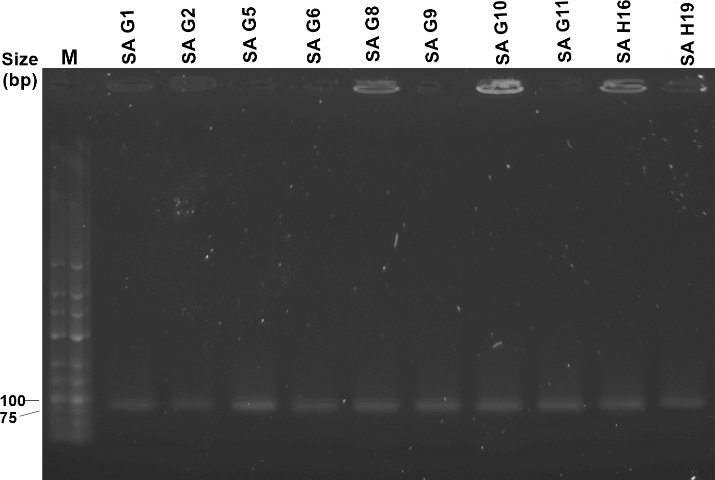


**Supplementary Fig. 13** Agarose gel electrophoresis of the PCR amplified products of *icaD* gene of *S. aureus* clinical isolates. Lane **M** indicates the low-range DNA ladder (Fermentas, USA). A 78 bp amplified product indicates a positive result


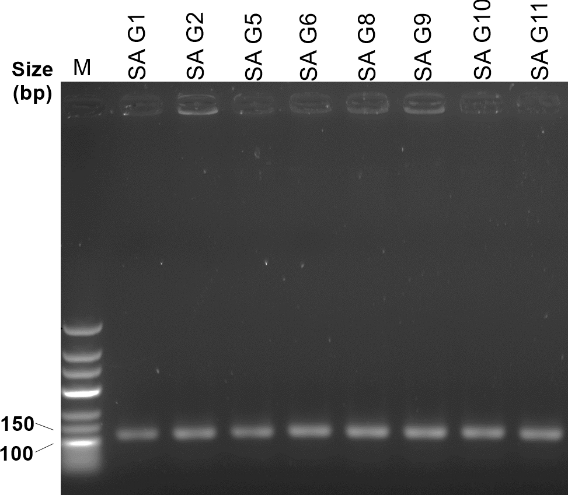

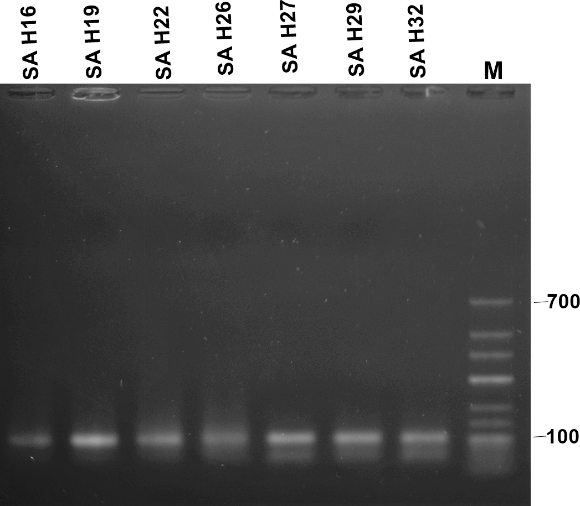


**Supplementary Fig. 14** Agarose gel electrophoresis of the PCR amplified products of *icaR* gene of *S. aureus* clinical isolates. Lane **M** indicates the low-range DNA ladder (Fermentas, USA). A 102 bp amplified product indicates a positive result


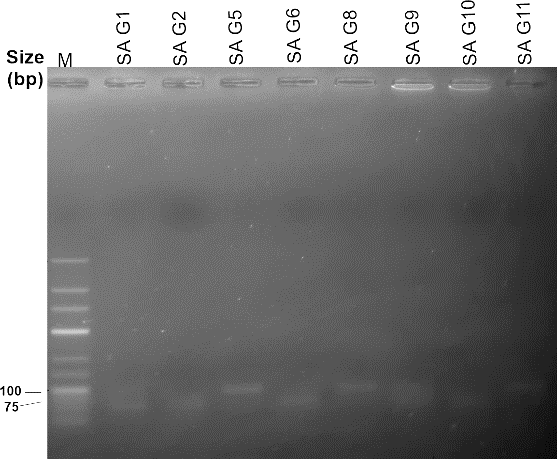

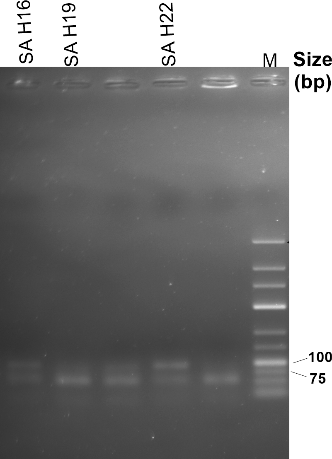

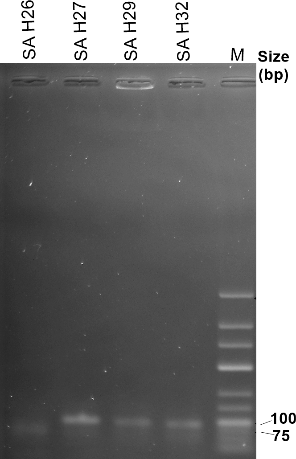


**Supplementary Fig. 15** Agarose gel electrophoresis of the PCR amplified products of *fnaA* gene of *S. aureus*

clinical isolates. Lane **M** indicates the low-range DNA ladder (Fermentas, USA).


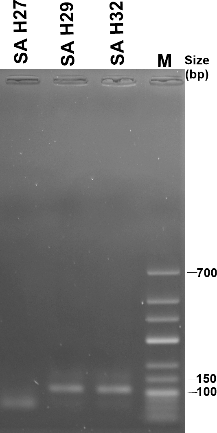

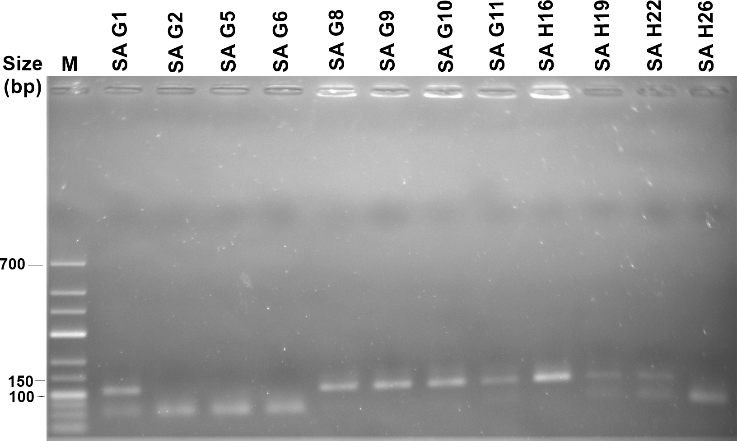


**S. 16.** Agarose gel electrophoresis of the PCR amplified products of *fnaB* gene of *S. aureus* clinical isolates. Lane **M** indicates the low-range DNA ladder (Fermentas, USA).


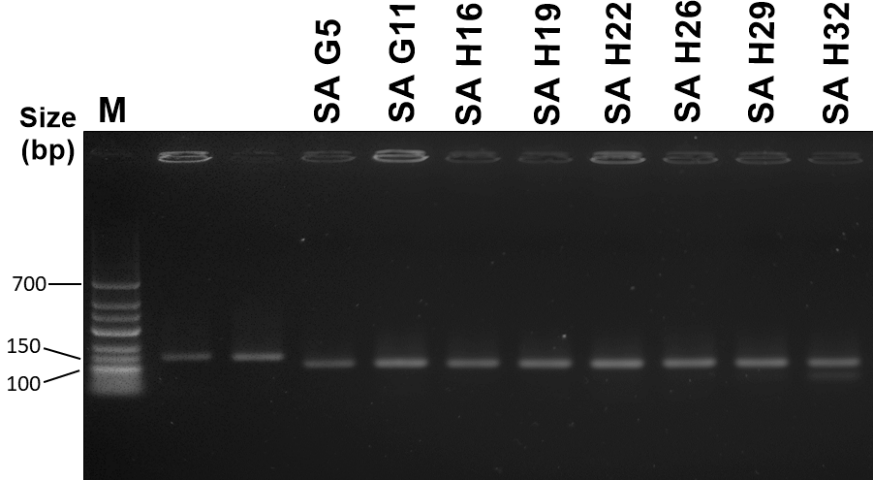


**Supplementary Fig. 17** Agarose gel electrophoresis of the PCR amplified products of *cna* gene of *S. aureus* clinical isolates. Lane **M** indicates the low-range DNA ladder (Fermentas, USA). A presence of 91-140 bp amplified product indicates a positive result


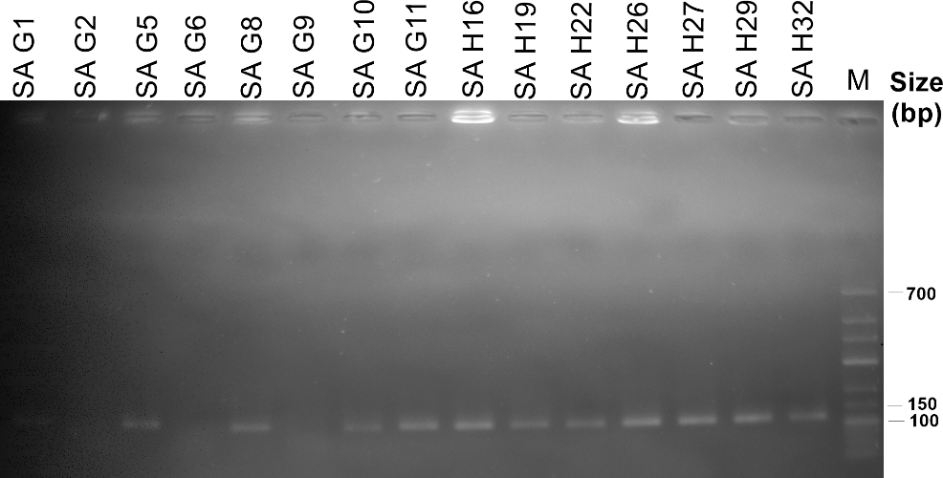


**Supplementary Fig. 18** Agarose gel electrophoresis of PCR amplified products of *clfA* gene of *S. aureus* clinical isolates. Lane **M** indicates the low-range DNA ladder (Fermentas, USA). A 115 bp amplified product indicates a positive result


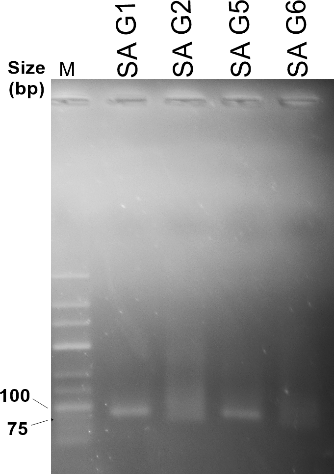

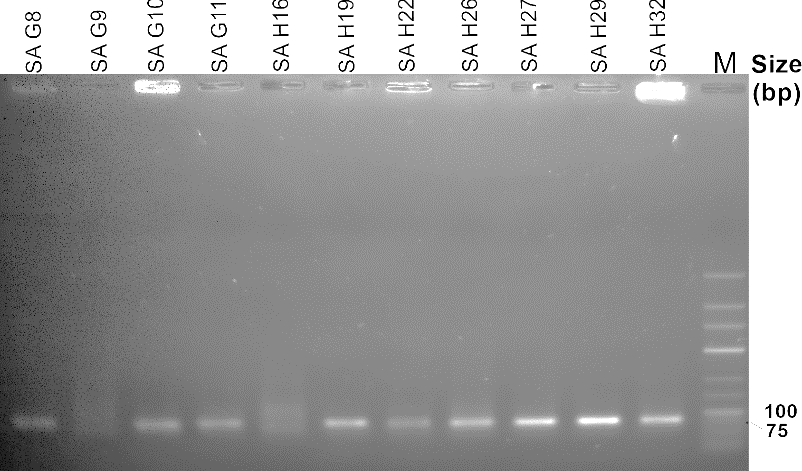


**Supplementary Fig. 19** Agarose gel electrophoresis of PCR amplified products of *clfB* gene of *S. aureus* clinical isolates. Lane **M** indicates the low-range DNA ladder (Fermentas, USA). A 77 bp amplified product indicates a positive result


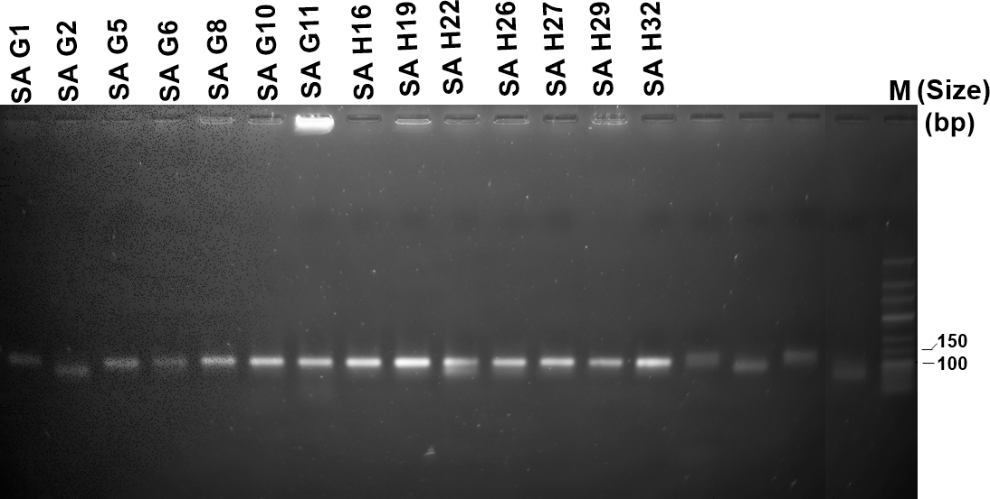


**Supplementary Fig. 20** Agarose gel electrophoresis of PCR amplified products of *ebps* gene of *S. aureus* clinical isolates. Lane **M** indicates the low-range DNA ladder (Fermentas, USA). A ~100 bp amplified product indicates a positive result.

**Supplementary Table 1.** Quantification of *S. aureus* biofilm-forming ability

| **Strain ID** | **Absorbance at 540nm** | **Category** |
| --- | --- | --- |
| SA G1 | 0.088±0.012 | Non |
| SA G2 | 0.092±0.015 | Non |
| SA G6 | 0.085±0.018 | Non |
| SA H16 | 0.066±0.016 | Non |
| SA G8 | 0.128±0.032 | Moderate |
| SA G9 | 0.160±0.009 | Moderate |
| SA G10 | 0.157±0.062 | Moderate |
| SA H19 | 0.142±0.011 | Moderate |
| SA H26 | 0.122±0.037 | Moderate |
| SA H32 | 0.133±0.051 | Moderate |
| SA G5 | 0.604±0.10 | Strong |
| SA G11 | 0.442±0.013 | Strong |
| SA H22 | 0.490±0.150 | Strong |
| SA H27 | 0.690±0.066 | Strong |
| SA H29 | 0.684±0.075 | Strong |
| ATCC25923 | 0.272±0.039 | Moderate |

**Supplementary Table 2.** Primers used for the detection of biofilm-associated genes

| **Gene name** | **Primer Sequence (5´- 3´)** | **Amplicon Size (bp)** |
| --- | --- | --- |
| *icaA* | TTTCGGGTGTCTTCACTCTATTT | 141 |
|  | TGGCAAGCGGTTCATACTT |  |
| *icaB* | ACCGGCAACTGGGTTTATT | 137 |
|  | GCAAATCGTGGGTATGTGTTTC |  |
| *icaC* | GCGTTAGCAAATGGAGACTATTG | 100 |
|  | GCGTGCAAATACCCAAGATAAC |  |
| *icaD* | AGCCCAGACAGAGGGAATA | 78 |
|  | ACGATATAGCGATAAGTGCTGTC |  |
| *icaR* | GCTGTTTCTTGAAAGTTGGTATTTG | 102 |
|  | AGTAGCGAATACACTTCATCTTTG |  |
| *fnbA* | AACAATCTTAGGTACGGCATTAGA | 89 |
|  | TCTTGTCCCATCCCAACAAC |  |
| *fnbB* | GTAGAGGAAAGTGGGAGTTCAG | 105 |
|  | TGTCGCGCTGTATGATTGT |  |
| *ebpS* | GTGGCATGGCCAAAGTATTG | 77 |
|  | CATGCCTCCAAATATCGCTAATG |  |
| *clfA* | CACAACAGGAAACGACACAATC | 115 |
|  | TGAGTTGTTGCCGGTGTATTA |  |
| *clfB* | CACAAACAGTGCGAATGTAGATAG | 77 |
|  | CTGGCTCTGTTGTAGTGGTATT |  |
| *cna* | CAGGTGGGTCAAGCAGTTATTA | 91 |
|  | CTGCAAATCCCGAAACATCAC |  |

**Supplementary Table 3.** Typing of *coa* gene and *Hae*III RFLP patterns of *S. aureus* strains

| **Strain ID** | **Detected *coa***  **amplicon size (bp)** | **RFLP pattern**  **(approx bp)** | **Pattern code** | **DI of *coa* PCR typing** | ***DI of *coa*-PCR-RFLP typing** |
| --- | --- | --- | --- | --- | --- |
| SA H19 | 550 | 400+150 | C1 | **0.8381** | **0.9619** |
| SA H26 | 550 | 400+150 | C1 |  |  |
| SA G6 | 550 | 405+145 | C2 |  |  |
| SA H29 | 550 | 200+150+100+90 | C3 |  |  |
| SA G11 | 600 | 250+200+150 | D |  |  |
| SA G1 | 650 | 220+190+150+90 | A |  |  |
| SA G2 | 650 | 220+190+150+90 | A |  |  |
| SA G9 | 660 | 505+155 | E |  |  |
| SA G5 | 700 | 230+200+170+90 | B1 |  |  |
| SA H16 | 700 | 600+150 | B2 |  |  |
| SA H22 | 700 | 600+150 | B2 |  |  |
| SA H27 | 700 | 450+150+100 | B3 |  |  |
| SA H32 | 700 | 450+150+100 | B3 |  |  |
| SA G8 | 740 | 590+150 | F |  |  |
| SA G10 | 800 | 250+200+150+100 | G |  |  |

***DI**-Discriminatory index

**Supplementary Table 4.** Typing of *spa* gene polymorphism and distribution of different repeats and types of *S. aureus* strains

| **Strain ID** | **Detected *spa* amplicon size (bp)** | **Repeats Succession** | **Kreiswirth IDs** | ***spa*-type** | ***DI of *spa*-PCR typing** |
| --- | --- | --- | --- | --- | --- |
| SA G1 | 440 | 11-19-21-12-21-17-34-24-34-22-25 | YHFGFMBQBLO | t051 | **0.9429** |
| SA G2 | 500 | 21-17-34-24-34-22-24-34-22-33-25 | FMBQBLQBLPO | UK |  |
| SA G5 | 480 | 08-39-34 | XE3B | t1011 |  |
| SA G6 | 440 | 26-17-16 | TMK | t535 |  |
| SA G8 | 400 | 26-17-20-17-12-17-17-16 | TMDMGMMK | t003 |  |
| SA G9 | 460 | 07-23-21-16-16-33-21-16-33-13 | UJFKKPFKPE | t175 |  |
| SA G10 | 380 | 04-20-17-20-17-25-34 | ZDMDMOB | t437 |  |
| SA G11 | 460 | 11-19-12-21-17-34-24-34-22-25 | YHGFMBQBLO | t008 |  |
| SA H16 | 355 | 26-23-17-12-17-16 | TJMGMK | t062 |  |
| SA H19 | 355 | 26-23-17-12-17-16 | TJMGMK | t062 |  |
| SA H22 | 460 | 26-23-17-34-17-82-17-12-17-16 | TJMBM[r82] MGMK | t2164 |  |
| SA H26 | 560 | 26 | T1 | t458 |  |
| SA H27 | 550 | 26-23-23-13-23-31-29-17-25-17-25-16-28 | TJJEJNF2MOMOKR | t379 |  |
| SA H29 | 430 | 11-19-12-21-17-34-24-34-22-25 | YHGFMBQBLO | t008 |  |
| SA H32 | 550 | 26-23-23-13-23 | TJJEJ | t1258 |  |

***DI**-Discriminatory index; **UK**- Unknown
